# Hierarchical micro-/nanostructured hydroxyapatite scaffolds promote osteoporotic bone regeneration via activation of hedgehog and HIF-1α signaling

**DOI:** 10.1016/j.bioactmat.2026.01.049

**Published:** 2026-02-05

**Authors:** Rui Zhao, Jiayi Chen, Yongjia Li, Hui Qian, Xiangdong Zhu, Grazia Raucci Maria, Luigi Ambrosio, Xiao Yang, Xingdong Zhang

**Affiliations:** a Department of Laboratory Medicine, School of Medicine, Jiangsu University, Zhenjiang, 212013, China; b National Engineering Research Center for Biomaterials, Sichuan University, Chengdu, 610064, China; c Institute of Polymers, Composites and Biomaterials, National Research Council, Naples, 80072, Italy

**Keywords:** Nano hydroxyapatite, Micro-/nano-structured bioceramics, Osteogenesis polarity, Osteoporotic bone regeneration, Hedgehog signaling

## Abstract

Osteoporotic bone defects remain a major clinical challenge due to impaired osteogenesis, disrupted angiogenesis, and poor scaffold integration. To overcome these limitations, we developed hierarchical micro-/nanostructured hydroxyapatite (nwHA) scaffolds by integrating morphology-specific nanohydroxyapatite (nHA) onto whisker-reinforced hydroxyapatite (wHA) scaffolds. This modular strategy decouples mechanical strength from interfacial bioactivity, enabling programmable topographical control. Five distinct nHA morphologies were used to functionalize wHA scaffolds, which were systematically evaluated both in vitro and in osteoporotic rat models. Among them, nanofiber-coated scaffolds (nwHA1) significantly enhanced bone volume fraction, mineral apposition rate, mechanical strength, and neovascularization. Histological analysis identified three distinct ossification patterns—type I (wall-penetrating), type II (surface-appositional), and a hybrid endochondral–intramembranous mode—whose distribution varied with nHA morphology and the local microenvironment. Mechanistically, nwHA1 activated canonical Hedgehog signaling and upregulated HIF-1α in both MSCs and HUVECs, thereby promoting coordinated osteogenic and angiogenic responses. Pharmacological inhibition with cyclopamine, as well as siRNA-mediated knockdown of GLI1 or HIF-1α, significantly attenuated these pro-osteoangiogenic markers, confirming functional crosstalk between Hedgehog and hypoxia signaling pathways in response to scaffold-induced topographic cues. These findings establish nHA morphology as a critical topographical regulator of bone regeneration and provide a versatile platform for designing adaptive bioceramics tailored to osteoporotic bone repair.

## Highlights

• Morphology-specific nHA-functionalized wHA scaffolds enhance osteogenesis and angiogenesis in osteoporotic bone defects.
• Nanofiber-coated nwHA1 scaffolds enhance bone regeneration and angiogenesis via Hedgehog-mediated osteoinduction.
• Three ossification modes (Type I, Type II, hybrid) are induced, guided by nHA topography and defect microenvironment.
• Hedgehog pathway activation upregulates SHH and GLI1, driving MSC differentiation and matrix mineralization.
• Nanoscale topography offers a programmable platform for adaptive bioceramics in osteoporotic bone repair.

## Graphical abstract

**Figure:**
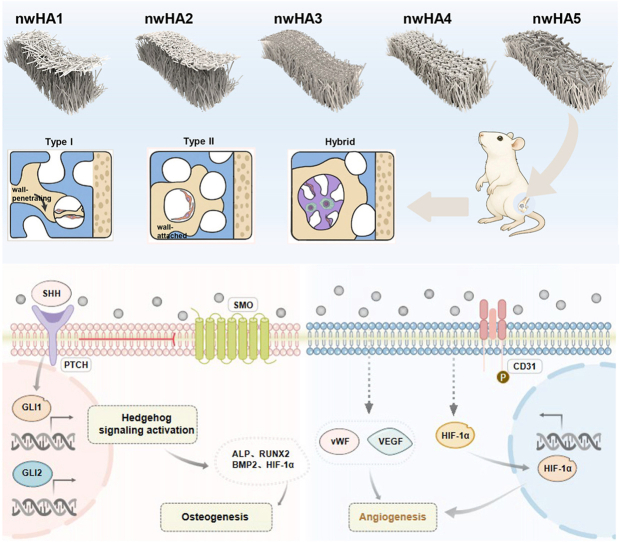

## 1 Introduction

Osteoporosis is a chronic degenerative disease characterized by reduced bone mass and microarchitectural deterioration, resulting in increased skeletal fragility and elevated fracture risk [1,2]. Osteoporotic fractures, increasingly prevalent in aging populations, are often associated with delayed healing, frequent nonunion, and substantial risks of disability and mortality, imposing a significant global health and socioeconomic burden [3]. Repairing osteoporotic bone defects remains clinically challenging, as compromised bone quality impairs mechanical integrity and disrupts the osteogenic microenvironment, hindering effective regeneration [4,5]. Moreover, excessive bone resorption and diminished osteogenic potential weaken implant anchorage and dysregulate local signaling cascades, limiting the efficacy of conventional interventions such as internal fixation [6]. Consequently, therapeutic strategies must concurrently address biomechanical instability and defective bone remodeling, underscoring the urgent need for advanced biomaterials to promote osteogenesis and restore homeostatic bone regeneration. Effective regeneration of osteoporotic bone requires biomaterials that recapitulate the hierarchical architecture and biochemical cues of native bone to guide cellular behavior and reestablish balanced tissue remodeling [7,8].

Hydroxyapatite (HA), a bioceramic that closely mimics the mineral phase of native bone, exhibits excellent osteoconductive and osteoinductive properties [9,10]. It promotes apatite-layer formation, facilitates ionic exchange with host tissues, and drives progenitor cell differentiation toward osteoblasts. However, in osteoporotic bone—where impaired mechanosensitivity and reduced tissue compliance disrupt force transmission—conventional HA scaffolds often fail to provide the necessary mechanical cues to activate osteogenic signaling, thereby limiting their regenerative potential [11,12]. Micro-/nanostructured HA scaffolds overcome these limitations by recapitulating the nanoscale topography of bone, improving mechanical integrity, and enhancing mechanotransduction to activate key developmental signaling pathways [[13], [14], [15]]. For example, Cui et al. showed that micro-/nanostructured HA bioceramics promote osteogenesis in osteoporotic conditions by stimulating autophagic activity in bone marrow-derived mesenchymal stem cells from ovariectomized rats. This enhances cell adhesion, drives osteogenic lineage commitment, and improves bone regeneration in calvarial defect models [16]. Notably, nanoscale topographic features, such as rods, act as potent bioinstructive cues that modulate mechanosensitive pathways and direct stem cell fate [17,18]. Among these pathways, the Hedgehog signaling cascade—essential for skeletal development—is strongly activated by nanostructured scaffold surfaces. Such topographies upregulate Sonic Hedgehog (SHH), Smoothened (SMO), and Glioma-associated oncogene 1 (GLI1) expression, thereby promoting osteoprogenitor differentiation and ossification, even in the absence of exogenous cytokines [[19], [20], [21]]. These findings indicate that nanotopographies can restore Hedgehog signaling in mesenchymal stem cells to counteract osteoporotic signaling deficits.

To overcome the mechanotransductive limitations of conventional HA in osteoporotic bone repair, whisker-reinforced HA (wHA) bioceramics provide superior mechanical performance. Their interlocked single-crystalline whiskers deliver up to 3.4-fold greater compressive strength [22]. However, the high crystallinity and dense microstructure of wHA limit surface bioactivity, thereby restricting osteoprogenitor adhesion and osteogenic signaling. Recent advances have introduced hollow-tube whisker architectures to better balance mechanical strength and biological performance. These designs increase specific surface area, promote M2 macrophage polarization, and stimulate angiogenesis through activation of the hypoxia-inducible factor 1-alpha (HIF-1α) pathway [[23], [24], [25], [26]]. The HIF-1α pathway, a key regulator of angiogenesis, supports endothelial cell migration and osteogenic differentiation via endothelial cell–mesenchymal stem cell crosstalk, as demonstrated in nanohydroxyapatite (nHA) co-culture studies [27]. Nevertheless, these strategies mainly focus on bulk microstructure modification, offering limited control over surface nanotopography and programmable biofunctionality. In our previous work, we pioneered micro-/nano-hybrid HA scaffolds by assembling rod-shaped nanoparticles onto wHA substrates, creating bioactive topographies without sacrificing mechanical integrity [22]. These hybrid scaffolds enhanced bone regeneration, immune modulation, and structural integration in beagle segmental defect models. Building on this, we further showed that such scaffolds promote osteogenesis in osteoporotic femoral defects by upregulating FGF23 through JAK2 signaling activation, underscoring the potential of micro-/nanostructured interfaces to modulate osteoblast behavior under pathological conditions [15]. Additionally, our micro-/nano-hybrid wHA bioceramics with releasable rod-shaped nHA stimulated osteoporotic osteoblast proliferation and differentiation in vitro and facilitated bone regeneration in osteoporotic rat femoral defects [28]. Scaffolds with optimized nHA loading induced uniform, mature ossification, demonstrating their tunability for osteogenesis in osteoporotic environments. Despite these advances, the influence of diverse nHA morphologies—such as fibers, spheres, or clusters—on mechanotransduction and stem cell lineage commitment remains largely unexplored.

**Fig. 1:**
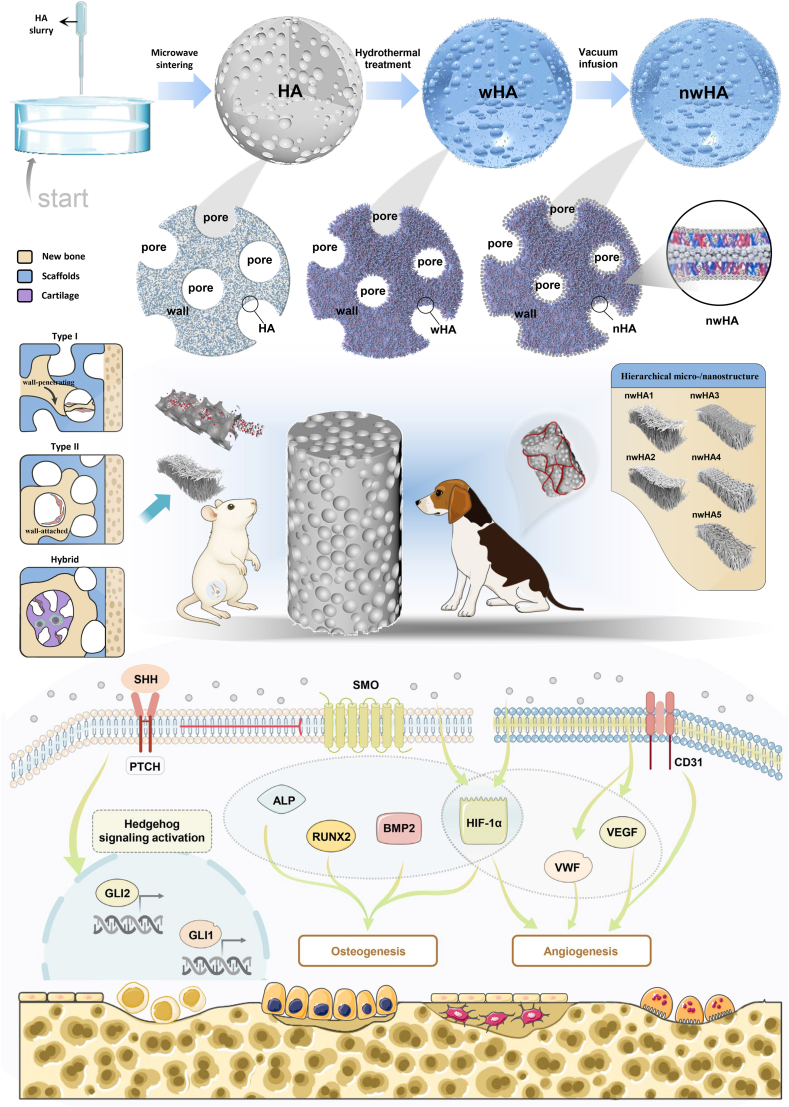
Diagram illustrating the preparation and proposed mechanism of action for nwHA scaffolds. Schematic showing how hierarchical micro-/nanostructured nwHA topographies enhance the osteogenic microenvironment in osteoporotic femoral defects. These topographies promote osteoprogenitor adhesion, vascular infiltration, and morphology-driven ossification modes—including wall-penetrating (type I), surface-appositional (type II), and hybrid patterns—thereby guiding context-specific intramembranous or endochondral bone regeneration.

To bridge this gap, we developed a hierarchical bioceramic platform by integrating hydrothermally synthesized nHA with five distinct morphologies onto wHA scaffolds via vacuum infusion (Fig. 1). This modular strategy enables independent tuning of nanoscale surface topography without compromising scaffold strength or porosity, effectively decoupling interfacial bioactivity from bulk mechanical architecture. Unlike prior studies that typically examined a single nHA morphology or relied on unreinforced ceramics, we systematically investigated how distinct nHA geometries regulate mesenchymal stem cell differentiation, angiogenic activation, and ossification dynamics specifically in osteoporotic microenvironments. Although nanotopographical cues are well known to modulate mechanotransduction and cell–matrix interactions, their precise roles under osteoporotic conditions remain poorly defined—a critical gap this study aims to address. We evaluated scaffold performance through in vitro osteogenic and angiogenic assays, supported by molecular pathway analyses. In vivo regenerative efficacy was assessed in osteoporotic rat femoral defects at 8 weeks post-implantation using micro-computed tomography, histomorphometry, and nanoindentation. By correlating surface morphology with structural, cellular, and molecular outcomes, this work establishes a mechanistic framework for the rational design of topography-programmable bioceramics tailored to osteoporotic bone repair.

## 2 Results

### 2.1 Synthesis and characterization of morphology-specific nHA

**Fig. 2:**
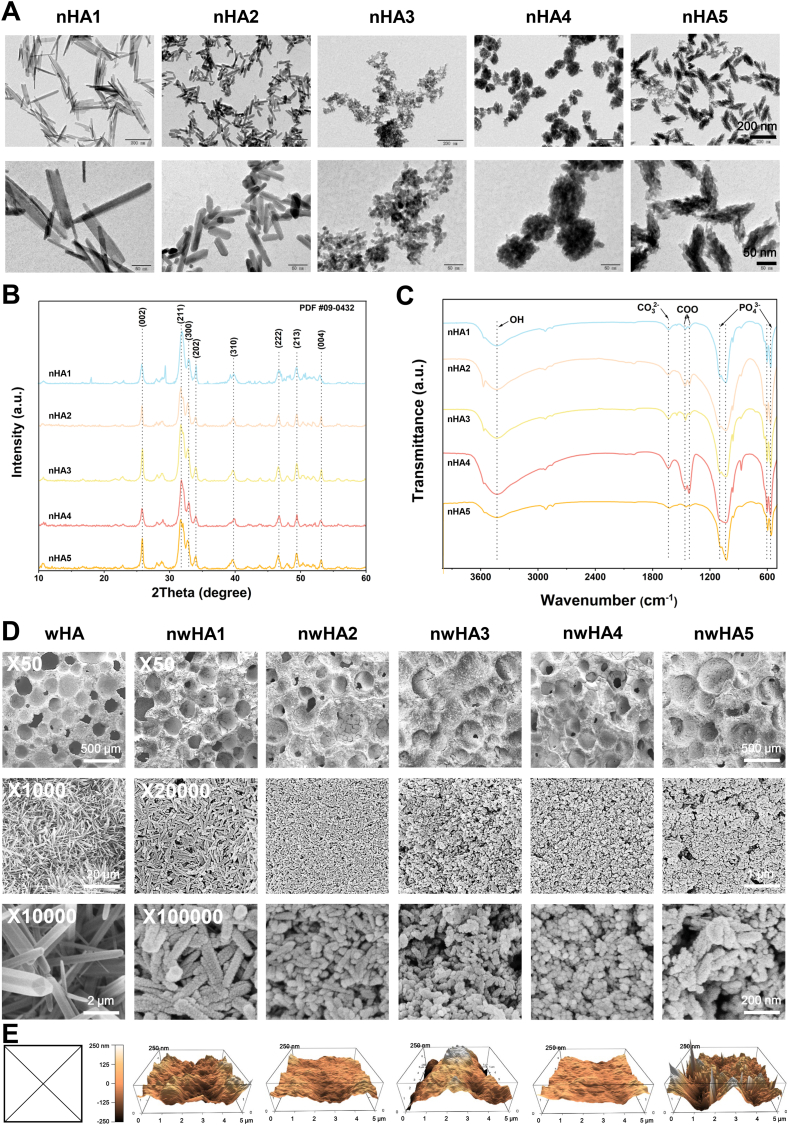
Characterization of morphology-specific nHA and nwHA scaffold surface architecture. (A) TEM images of five nHA morphologies, including nanofibers, nanorods, primary nanoparticles, raspberry-like nanospheres, and corn-like nanoaggregates, synthesized via tailored hydrothermal methods. (B) XRD patterns and (C) FTIR spectra of nwHA scaffolds. (D) Representative SEM images and (E) AFM-based surface roughness analysis performed on dense ceramic analogs fabricated using identical whiskerization and nHA-coating protocols. Direct AFM imaging was not feasible for wHA due to excessive vertical relief (>1000 nm), which exceeded the operational range of the instrument. As such, the far-left black-framed box marked with an “X” indicates imaging failure for the wHA group. The schematic highlights representative post-coating surface morphologies across groups and emphasizes relative topographical differences.

Morphology-specific nanohydroxyapatite (nHA) particles were synthesized by precisely tuning calcium/phosphate precursor systems to control nucleation and crystal growth kinetics. The colloidal properties of the as-prepared nHA were first evaluated by dynamic light scattering (DLS) (Supplementary Fig. S1). nHA1 and nHA2 displayed relatively larger hydrodynamic diameters (173.9 nm and 67.1 nm, respectively) and more negative zeta potentials (−17.0 mV and −12.7 mV), indicating better colloidal stability and dispersion. In contrast, nHA4 and nHA5 exhibited larger hydrodynamic sizes with less negative surface charges, consistent with their stronger aggregation tendency (Supplementary Table S1). Transmission electron microscopy (TEM) confirmed the formation of five distinct morphologies (Fig. 2A). nHA1 consisted of elongated nanofibers with an average length of 237.2 ± 47.3 nm and width of 17.8 ± 6.0 nm. nHA2 appeared as short nanorods (67.5 ± 16.2 nm in length × 17.3 ± 4.4 nm in width). nHA3 comprised ultrafine primary nanoparticles with a mean diameter of 9.4 ± 1.9 nm, showing low crystallinity and a high propensity for aggregation. nHA4 formed raspberry-like spherical assemblies (87.6 ± 7.0 nm in diameter), while nHA5 exhibited corn-like aggregates (134.5 ± 18.0 nm in length × 46.3 ± 3.5 nm in width). These results demonstrated a controlled transition from anisotropic single crystals to higher-order clustered structures by modulating precursor chemistry and synthesis conditions. X-ray diffraction (XRD) analysis revealed that all nHA variants displayed characteristic diffraction peaks of hexagonal hydroxyapatite (JCPDS No. 09–0432), confirming phase purity (Fig. 2B). nHA1 and nHA2 showed higher relative crystalline ordering than nHA3–nHA5, consistent with morphology-dependent differences in nanocrystalline domain ordering. Fourier-transform infrared (FTIR) spectroscopy further verified the chemical composition (Fig. 2C). All samples displayed typical phosphate vibration bands at 525–600 cm^−1^ and 865–1150 cm^−1^, along with a weak carbonate-related band at ∼1410–1470 cm^−1^, indicating minor carbonate incorporation typical of aqueous-synthesized, biomimetic apatite [10]. Inductively coupled plasma optical emission spectroscopy (ICP-OES) revealed morphology-dependent Ca/P molar ratios. nHA1 and nHA2 showed ratios of 1.61 and 1.58, respectively, close to the stoichiometric hydroxyapatite value (∼1.67), whereas nHA3, nHA4, and nHA5 exhibited lower ratios of 1.31, 1.32, and 1.43, respectively, consistent with calcium-deficient, low-crystallinity apatite phases commonly observed in biomimetic or rapidly precipitated systems.

### 2.2 Surface architecture and characterization of nwHA bioceramic scaffolds

Hierarchical nwHA bioceramic scaffolds were fabricated by vacuum-assisted infusion of morphology-tailored nHA onto whisker-reinforced hydroxyapatite (wHA) substrates, creating engineered nanotopographies while preserving the underlying whisker-reinforced framework. Scanning electron microscopy (SEM) imaging showed that uncoated wHA scaffolds featured an interconnected porous network of densely interwoven three-dimensional hydroxyapatite microwhiskers (Fig. 2D). Following nHA deposition, all nwHA groups exhibited compact nanosurfaces formed by particle aggregation, with coating architectures that reflected the distinct geometries and packing densities of the respective nHA particles: nanofibers (nwHA1), nanorods (nwHA2), primary nanoparticles (nwHA3), raspberry-like nanospheres (nwHA4), and corn-like nanoaggregates (nwHA5). Notably, nwHA5-coated scaffolds displayed pronounced surface irregularity, characterized by greater vertical height variations and more rugged hierarchical features, consistent with the aggregated nature of nHA5. Micro-CT morphometric analysis confirmed that nHA coatings did not significantly alter overall porosity or macropore size (∼200 μm), maintaining consistency in bulk scaffold architecture (Supplementary Fig. S2). Brunauer–Emmett–Teller (BET) surface area analysis revealed an increase in specific surface area (SSA) for all nHA-coated scaffolds relative to uncoated wHA, although no significant differences were observed among the different nwHA groups (Supplementary Fig. S3). To characterize nanoscale topography, atomic force microscopy (AFM) was performed. Due to the high vertical complexity of the macroporous scaffolds, AFM imaging and roughness analysis were conducted on flat ceramic analog substrates processed in parallel using the same hydrothermal whiskerization and nHA coating treatments. Anisotropic coatings (nwHA1 nanofibers and nwHA2 nanorods) produced comparatively smoother, flatter nanosurfaces, whereas the aggregated morphology (nwHA5 corn-like nanoaggregates) yielded the most irregular, topographically complex surfaces; nwHA3 (primary nanoparticles) and nwHA4 (raspberry-like nanospheres) showed intermediate profiles. Quantitative roughness parameters (Ra and Rq) were reported in Supplementary Table S2. Early-stage dissolution behavior was assessed by measuring ion release profiles in Tris-HCl buffer (pH 7.4) over 1–3 days. All groups showed controlled release of Ca^2+^ and PO_4_^3−^ within a narrow concentration range (0–0.4 μg/mL), with no evidence of burst release (Supplementary Fig. S4). Subtle morphology-dependent differences were observed: nwHA1 and nwHA2 displayed more gradual release profiles, whereas nwHA3 (and to a lesser extent nwHA4) exhibited slightly higher cumulative ion release. These variations align with the crystallinity and morphology of the nHA particles and provide a foundation for interpreting their differential biological performance.

### 2.3 Cell proliferation and osteogenic differentiation on bioceramic scaffolds

**Fig. 3:**
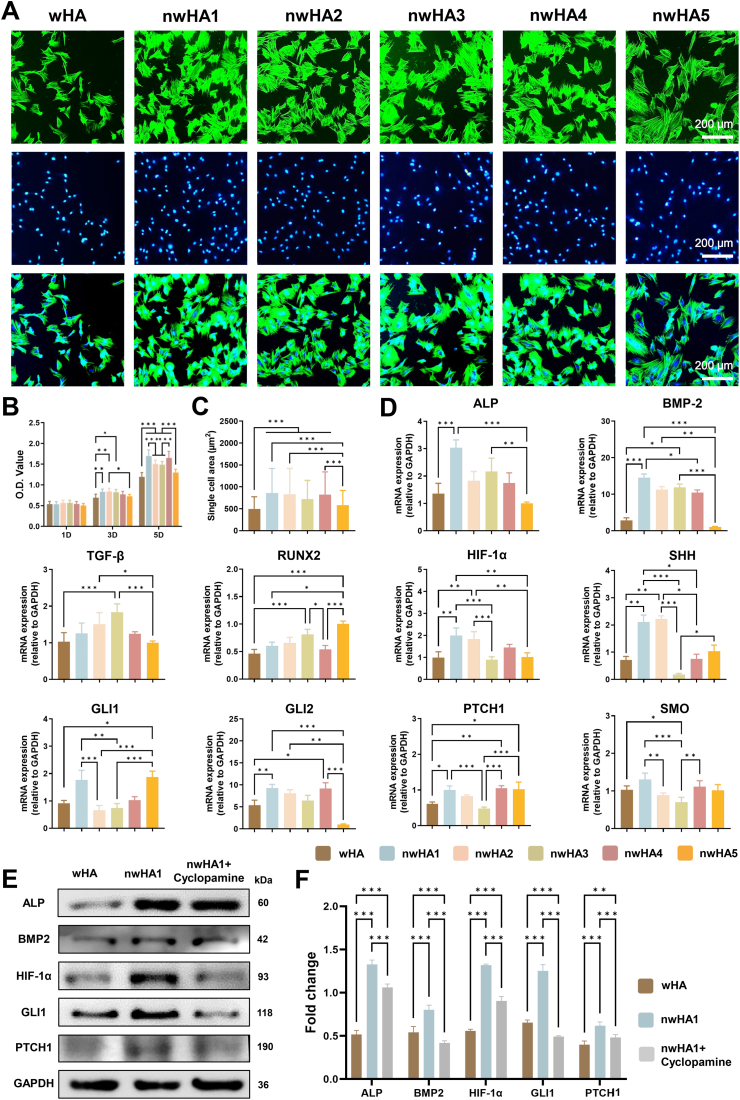
In vitro evaluation of biocompatibility and osteogenic activity of wHA and nwHA scaffolds using MSCs. (A) Fluorescence staining for F-actin (phalloidin-TRITC, green) and nuclei (DAPI, blue), showing enhanced cell spreading. (B) CCK-8 proliferation assay; data presented as mean ± SD (n = 8, ∗*p* < 0.05, one-way ANOVA with Tukey's post hoc test). (C) Quantification of single-cell area; data presented as mean ± SD (n ≥ 10, ∗*p* < 0.05, one-way ANOVA with Tukey's post hoc test). (D) qRT-PCR of osteogenic and Hedgehog-related markers (ALP, BMP2, Runx2, HIF-1α, SHH, PTCH1, SMO, GLI1, GLI2) at day 2; data presented as mean ± SD (n = 6, ∗*p* < 0.05, Kruskal–Wallis with Dunn's post hoc test, when applicable). (E) Western blot and (F) corresponding densitometric quantification of ALP, BMP2, HIF-1α, and GLI1 expression (n > 6, ∗*p* < 0.05, ∗∗*p* < 0.01, ∗∗∗*p* < 0.001, one-way ANOVA with Tukey's post hoc test).

The cytocompatibility and osteogenic potential of the nwHA bioceramic scaffolds were evaluated in vitro using mouse bone marrow-derived mesenchymal stem cells (MSCs) (Fig. 3A). Phalloidin/DAPI staining showed that MSCs adhered well and exhibited substantial spreading on all nwHA scaffolds, indicating stronger cell–material interactions compared with uncoated wHA (Fig. 3B). Cell viability, assessed by Cell Counting Kit-8 (CCK-8) assays, was significantly higher across all nwHA groups, demonstrating that the hierarchical surface topographies promoted MSC proliferation. Single-cell spreading areas were also significantly larger on nwHA scaffolds, particularly for nwHA1 and nwHA2, whereas nwHA5 showed spreading comparable to wHA (Fig. 3C). This enhanced spreading on nwHA1 and nwHA2 is attributable to their finer nanoscale features, which provide increased local contact points and facilitate early cytoskeletal organization. Quantitative analysis of cell morphology further revealed stronger cytoskeletal alignment and greater shape anisotropy in MSCs cultured on nwHA1 and nwHA2 scaffolds, as evidenced by clustered FeretAngle distributions and elevated MaxFeret/MinFeret ratios. In contrast, MSCs on wHA and nwHA5 displayed broader angular dispersion and reduced shape anisotropy (Supplementary Fig. S5). These findings suggest that the nanotopographies of nwHA1 and nwHA2 modulate MSC polarity, an early regulator of subsequent extracellular matrix organization and osteogenic differentiation. Extract-based cytocompatibility assays confirmed that all scaffold extracts were non-toxic to MSCs, with extracts from nwHA1 and nwHA2 significantly enhancing viability relative to wHA. Live/dead staining further supported these observations (Supplementary Fig. S6). Osteogenic staining at day 7 also revealed morphology-dependent differences in early and late-stage osteogenic responses (Supplementary Fig. S7).

At the molecular level, quantitative real-time PCR (qRT-PCR) demonstrated substantial upregulation of osteogenic markers in MSCs cultured on nwHA1 scaffolds compared with wHA, including alkaline phosphatase (ALP; 2.2-fold), bone morphogenetic protein 2 (BMP2; 5.0-fold), and hypoxia-inducible factor 1-alpha (HIF-1α; 2.0-fold) (Fig. 3D). nwHA3 induced moderate increases in RUNX2, BMP2, and TGF-β expression, highlighting morphology-specific modulation of osteogenic gene profiles.

Analysis of Hedgehog signaling pathway markers showed significantly elevated sonic hedgehog (SHH) expression on nwHA1 and nwHA2 scaffolds (2.9-fold and 3.1-fold higher than wHA, respectively). nwHA1 also upregulated key components, including smoothened (SMO; 1.3-fold), glioma-associated oncogene 1 (GLI1; 1.9-fold), GLI2 (1.7-fold), and patched 1 (PTCH1; 1.6-fold), while nwHA2 and nwHA3 showed similar but less pronounced activation trends. Western blot analysis validated increased protein levels of ALP, BMP2, HIF-1α, and GLI1 in the nwHA1 group (Fig. 3E and F). Pharmacological inhibition with cyclopamine significantly suppressed these markers, confirming the involvement of Hedgehog signaling in nwHA-mediated osteogenic activation. To explore the mechanistic interplay between Hedgehog and hypoxia-related signaling, reciprocal siRNA knockdown experiments were performed in MSCs cultured on nwHA1 scaffolds. GLI1 silencing markedly reduced both GLI1 and HIF-1α expression at mRNA and protein levels, indicating that Hedgehog signaling contributes to HIF-1α activation in response to scaffold-induced cues (Supplementary Fig. S8). Conversely, HIF-1α knockdown attenuated GLI1 expression, suggesting bidirectional crosstalk between the two pathways. Functionally, knockdown of either GLI1 or HIF-1α significantly suppressed the expression of key osteogenic markers (ALP, RUNX2, and OCN) at the mRNA and protein levels, demonstrating that the Hedgehog–HIF-1α axis is essential for the topography-driven osteogenic program induced by nwHA1 (Supplementary Fig. S9).

### 2.4 Pro-angiogenic effects of nwHA scaffolds on HUVECs

**Fig. 4:**
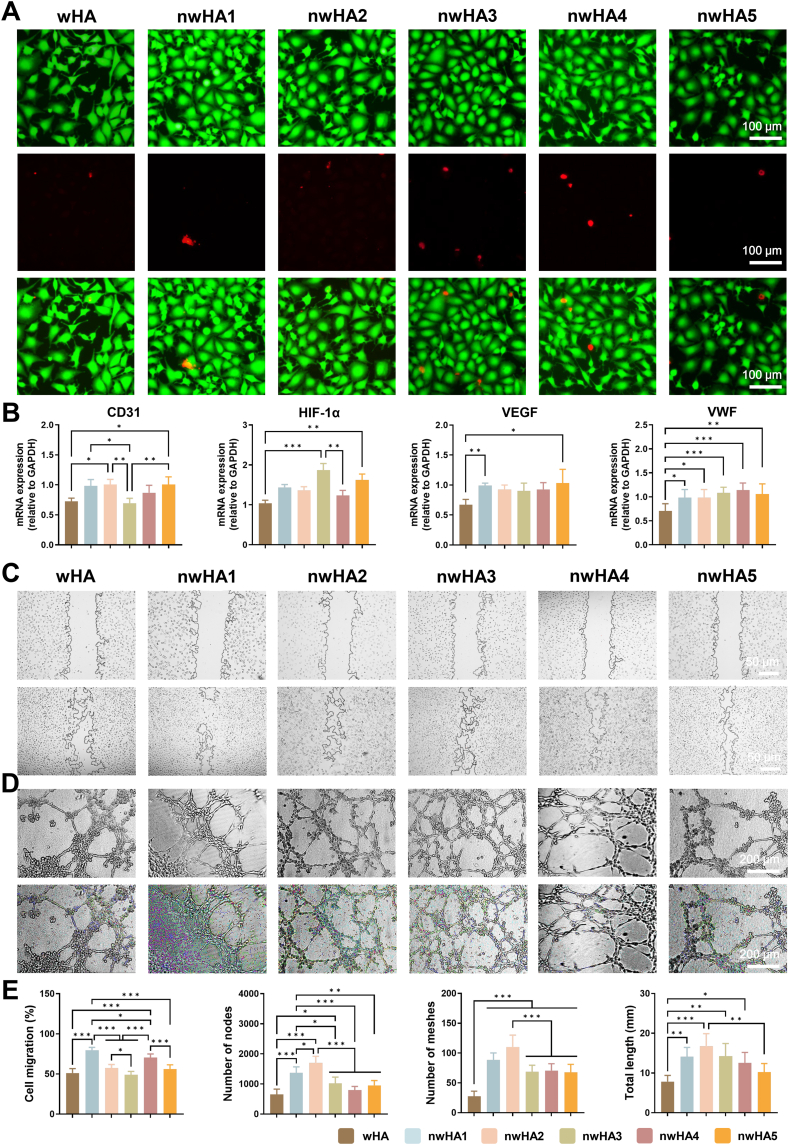
In vitro angiogenic activity of wHA and nwHA scaffolds using HUVECs. (A) Live/dead staining of HUVECs on scaffolds (calcein-AM: green; PI: red). (B) qRT-PCR analysis of angiogenic markers (CD31, VEGF, VWF, HIF-1α) (n = 6). Scale bar: 100 μm. (C) Scratch wound healing assay assessing HUVEC migration in a semi-contact configuration with bioceramic scaffolds placed adjacent to—but not in contact with—the scratch area; images acquired at 0 h and 24 h. Scale bar: 50 μm. (E) Quantification of total tube length, mesh number, and node count (n ≥ 6). All values are presented as mean ± SD; ∗*p* < 0.05, ∗∗*p* < 0.01, ∗∗∗*p* < 0.001 (ANOVA with Tukey's post hoc test).

**Fig. 5:**
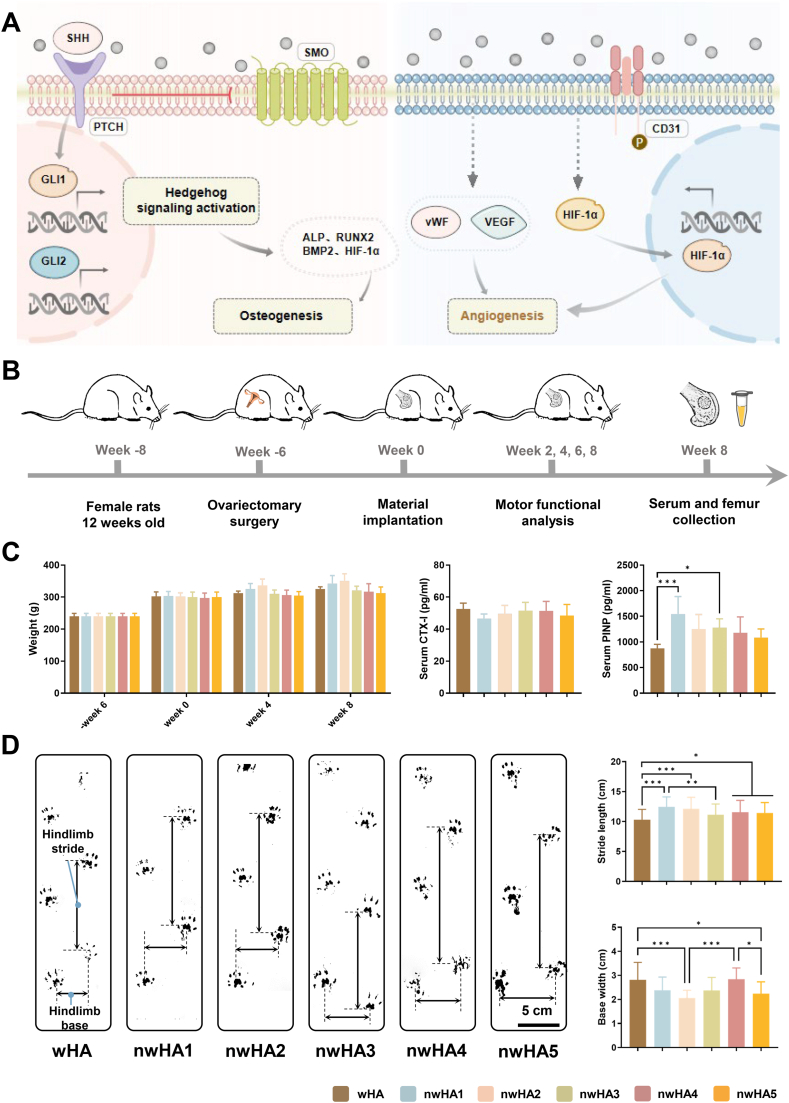
In vivo bone regeneration and functional recovery in osteoporotic rat femoral defects. (A) Schematic illustrating nwHA1-induced osteogenesis via Hedgehog activation in MSCs and pro-angiogenic effects in HUVECs through HIF-1α upregulation. (B) Surgical implantation of wHA and nwHA scaffolds (nwHA1–nwHA5) into distal femoral defects; evaluation at 8 weeks. (C) Body weight and serum markers (CTX-I, PINP) at 8 weeks; data shown as mean ± SD (n = 5; ∗*p* < 0.05, ∗∗∗*p* < 0.001, Kruskal–Wallis with Dunn's post hoc test). (D) Gait analysis quantifying stride length and base width as indicators of functional recovery; data shown as mean ± SD (n ≥ 8; ∗*p* < 0.05, ∗∗*p* < 0.01, ∗∗∗*p* < 0.001; one-way ANOVA with Tukey's post hoc test).

The pro-angiogenic potential of the nwHA scaffolds was assessed using human umbilical vein endothelial cells (HUVECs) cultured in direct contact with bioceramic discs. CCK-8 assays and live/dead staining demonstrated significantly improved endothelial viability and adhesion across all nwHA groups compared with uncoated wHA controls (Fig. 4A and Supplementary Fig. S10). qRT-PCR revealed morphology-dependent upregulation of key angiogenic genes, most notably in the nwHA1 and nwHA2 groups. Expression of CD31, VEGF, VWF, and HIF-1α was increased by approximately 1.3–1.4-fold relative to wHA (Fig. 4B), indicating enhanced endothelial activation driven by nanoscale topographical cues. To determine the role of HIF-1α in mediating these transcriptional responses, siRNA knockdown experiments were conducted with and without nwHA1 stimulation. In the absence of scaffold contact, HIF-1α knockdown resulted in minimal changes in CD31 and VWF expression, accompanied by a slight compensatory increase in VEGF (Supplementary Fig. S11). In contrast, under nwHA1 stimulation, HIF-1α silencing significantly suppressed VEGF, CD31, VWF, and HIF-1α expression, confirming that HIF-1α activation is scaffold-induced and essential for full endothelial transcriptional activation. Functional assays further corroborated these molecular findings. In scratch wound healing assays, HUVECs cultured in proximity to nwHA1 or nwHA2 scaffolds exhibited significantly faster wound closure than those with wHA (Fig. 4C and Supplementary Fig. S12), with nwHA1 showing the greatest enhancement in migration at 24 h. In Matrigel tube formation assays, HUVECs preconditioned on nwHA scaffolds formed more extensive capillary-like networks compared with wHA (Fig. 4D). Quantitative analysis revealed significantly greater total tube length, node number, and mesh count for nwHA1 and nwHA2 (Fig. 4E), with nwHA1 exhibiting the strongest pro-angiogenic effect overall. To investigate potential involvement of Hedgehog signaling in endothelial responses, we analyzed expression of GLI1, PTCH1, and SHH. Unlike the robust Hedgehog activation observed in MSCs, these markers showed no consistent upregulation across scaffold groups (Supplementary Fig. S13), suggesting that endothelial responses were primarily mediated by HIF-1α rather than canonical Hedgehog signaling. These results demonstrate that nwHA scaffolds—particularly nwHA1 and nwHA2—promote endothelial activation, migration, and tubulogenesis in a morphology-dependent manner, with HIF-1α acting as a key scaffold-responsive regulator. A schematic summary of the proposed mechanism is provided in Fig. 5A.

### 2.5 In vivo bone regeneration and serum biomarker analysis

The regenerative performance of the nwHA scaffolds was evaluated in a critical-sized femoral defect model in osteoporotic rats (Fig. 5B). Scaffolds (wHA, nwHA1–nwHA5) were implanted, and outcomes were assessed at 8 weeks post-surgery. All animals tolerated the procedure well, with no signs of infection, wound dehiscence, or abnormal healing. Body weight remained stable across all groups throughout the study period (Fig. 5C). Serum biomarkers were analyzed to monitor systemic bone metabolism. The bone resorption marker C-terminal cross-linked telopeptide of type I collagen (CTX-I) showed no significant differences among groups, indicating that scaffold implantation did not substantially alter systemic bone resorption. In contrast, the bone formation marker procollagen type I N-terminal propeptide (PINP) was significantly elevated in the nwHA1 and nwHA3 groups compared with the wHA group, reflecting an enhanced anabolic response. Functional recovery was evaluated using gait analysis as a measure of limb use and biomechanical restoration. At 8 weeks, nwHA1, nwHA2, nwHA4, and nwHA5 groups exhibited significantly longer stride lengths than the wHA group, suggesting improved weight-bearing capacity (Fig. 5D). Notably, the nwHA2 group also displayed the smallest base width, indicating better limb alignment and postural stability. These functional improvements were evident from week 2 onward and persisted through week 8 (Supplementary Fig. S14), demonstrating the sustained regenerative benefits of nwHA1 and nwHA2. These in vivo results, together with the in vitro findings, show that morphology-specific nHA coatings significantly enhance bone regeneration in osteoporotic defects. Among the scaffold groups, nwHA1 and nwHA2 provided the most consistent advantages in functional recovery and systemic bone formation markers, underscoring the critical role of scaffold surface morphology in promoting osteoporotic bone repair.

### 2.6 Quantification of bone repair and osseointegration in osteoporotic defects

**Fig. 6:**
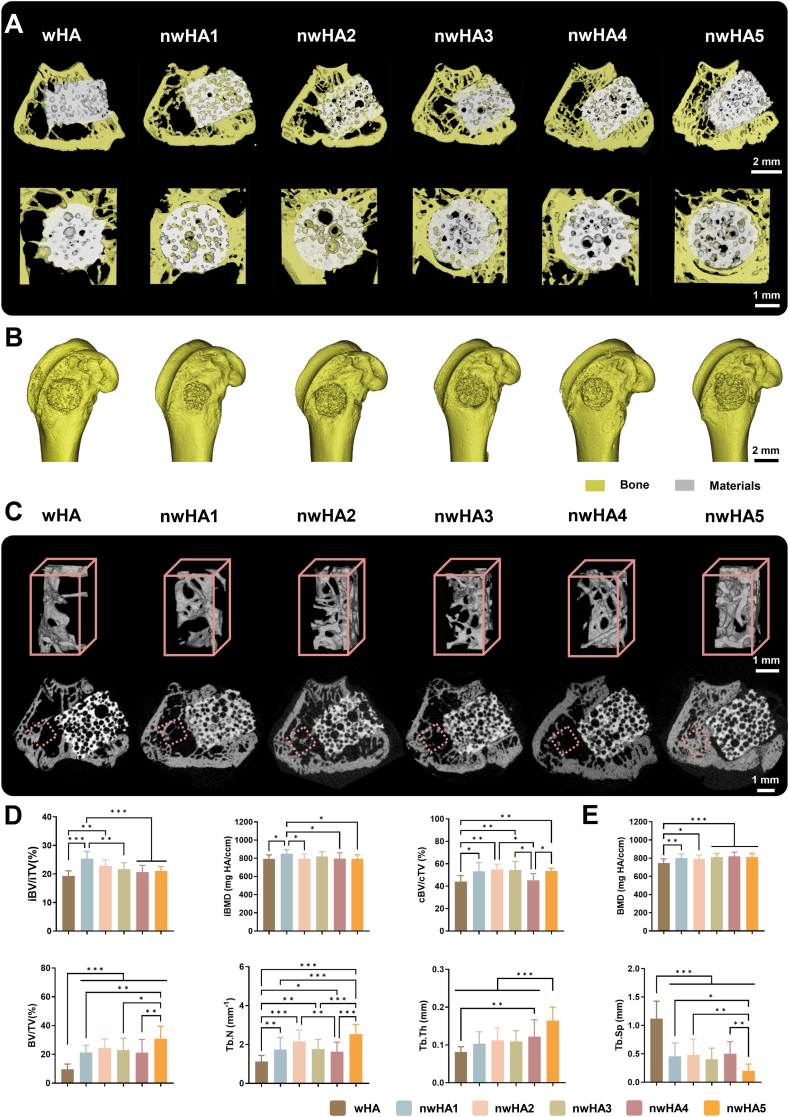
Micro-CT assessment of in vivo bone regeneration at 8 weeks. (A) 3D micro-CT reconstructions showing new bone formation and bone–scaffold interface (white: scaffold; yellow: bone); scale bar: 1 mm. (B) Whole femoral metaphysis reconstruction; scale bar: 2 mm. (C) Top: 3D renderings of trabecular ROI (red dotted box); bottom: corresponding 2D cross-sections at implant–bone interface; scale bar: 1 mm. (D) Quantification of intra-defect bone volume fraction (iBV/iTV), bone mineral density (iBMD), and concentric bone volume (cBV/cTV) within a 100 μm annular ROI; data are mean ± SD (n = 8; ∗*p* < 0.05, ∗∗*p* < 0.01, ∗∗∗*p* < 0.001). (E) Histomorphometric analysis of trabecular microarchitecture parameters including BMD, BV/TV, BS/BV, Conn.Dens, Tb.Th, Tb.Sp, and SMI; data as mean ± SD (n = 8; ∗*p* < 0.05, ∗∗*p* < 0.01, ∗∗∗*p* < 0.001).

Micro-computed tomography (micro-CT) revealed substantial new bone formation within osteoporotic femoral defects and along the scaffold-host interface in the nwHA groups (Fig. 6A and B). Three-dimensional reconstructions showed pronounced bone ingrowth in all nHA-coated scaffolds, with nwHA1 (nanofibers) and nwHA2 (nanorods) exhibiting clear osseous bridging from the defect margins toward the center and intimate bone–scaffold contact. In contrast, uncoated wHA scaffolds displayed limited intraporous bone formation and visible interfacial gaps, indicating impaired osseointegration under osteoporotic conditions. Early osseointegration was further quantified at 1 week by measuring bone formation within a defined 100 μm peri-implant gap, an established metric for initial interface integration [15,28]. All nHA-coated scaffolds showed significantly enhanced early mineralization compared with wHA, with nwHA1, nwHA2, and nwHA3 exhibiting the greatest interfacial bone deposition at this stage (Supplementary Fig. S15). To assess degradation–regeneration dynamics, we calculated the bone substitution rate (iBV/DV), where iBV is defined as the intradefect new-bone volume and DV as the degraded scaffold volume (DV = MV − RMV, with MV the initial scaffold volume and RMV the residual material volume at the endpoint). Although DV was comparable across groups, nwHA1 displayed the highest iBV/DV, indicating the most effective replacement of degraded scaffold with new bone (Supplementary Fig. S16). Consistent with this, nwHA1 also achieved significantly higher intraporous bone volume fraction (iBV/iTV), new bone mineral density (iBMD), and interface bone volume (cBV/cTV) relative to wHA (Fig. 6D), supporting its superior remodeling profile. Cross-sectional reconstructions highlighted abundant periscaffold bone adjacent to nwHA2 and nwHA5, with magnified views revealing continuous trabecular organization and compact bone-like structures (Fig. 6C). Quantitative analysis of peri-defect trabecular bone showed that nHA-coated scaffolds improved host bone architecture, as evidenced by increased bone volume fraction (BV/TV), higher trabecular number (Tb.N), and reduced trabecular separation (Tb.Sp) (Fig. 6E). Notably, nwHA5 exhibited the highest peri-scaffold BV/TV and trabecular thickness (Tb.Th), despite relatively weaker intra-scaffold osteogenesis. This observation indicates spatially distinct effects: the highly irregular surface of nwHA5 may limit internal cell organization and bone ingrowth, while local particle degradation or redistributed nHA fragments could enhance trabecular remodeling in the surrounding host bone. These findings highlight the dual influence of morphology-specific nHA: nanoscale surface architecture directs local cell–material interactions and intraporous regeneration, whereas degradation products may further modulate peri-scaffold host remodeling. Among the tested morphologies, nwHA1 and nwHA2 provided the most balanced performance, achieving robust intra-scaffold bone formation, effective osseointegration, and optimal degradation–regeneration coupling in osteoporotic defects.

### 2.7 Early-stage host response to ectopic implantation in skeletal muscle

To evaluate the initial host response in a bone-independent environment, uncoated wHA and nHA-coated scaffolds were implanted into the hindlimb musculature of healthy rats and retrieved at 10 days post-implantation. Hematoxylin and eosin (H&E) staining revealed robust peri-implant cellular infiltration in all groups, predominantly composed of macrophages, fibroblasts, lymphocytes, polymorphonuclear leukocytes, multinucleated giant cells, and erythrocytes. These cells were frequently organized around primitive vessel-like structures (Supplementary Fig. S17). Cellular infiltration into the internal macropores remained limited, consistent with the early stage of implantation. Among the scaffold groups, nwHA1 exhibited the most extensive peri-implant infiltration and the highest degree of intraporal cellular penetration, followed by nwHA2, nwHA3, nwHA4, and nwHA5. Uncoated wHA scaffolds showed the weakest cellular response. Immunohistochemical analysis of peri-implant regions (quantified in four standardized corner areas per section) demonstrated significant upregulation of GLI1 expression in nwHA1, nwHA2, nwHA3, and nwHA5 compared with nwHA4 and wHA, with nwHA4 showing intermediate levels. BMP2 expression was elevated in all nHA-coated scaffolds relative to wHA, with nwHA4 exhibiting the strongest signal, followed by nwHA1, nwHA2, nwHA3, and nwHA5—all significantly higher than uncoated wHA. HIF-1α expression was most prominent in nwHA5, followed by nwHA1 and nwHA4, with lower levels in nwHA2, nwHA3, and wHA. To assess early vascularization, immunofluorescence co-staining for CD31 and endomucin (Emcn) was performed. nwHA1, nwHA2 and nwHA3 showed abundant vessel-like structures in peri-implant tissue and increased numbers of CD31^+^Emcn^+^ capillaries, which are associated with enhanced angiogenesis and subsequent osteogenic coupling (Supplementary Fig. S18) [28]. These observations indicate that nanoscale surface topography rapidly influences tissue recruitment and signaling activation in ectopic implantation sites within the first 10 days, with morphology-dependent effects: nwHA1 most effectively promoted cellular infiltration and early vascularization.

### 2.8 Histological and micromechanical evaluation of bone regeneration

**Fig. 7:**
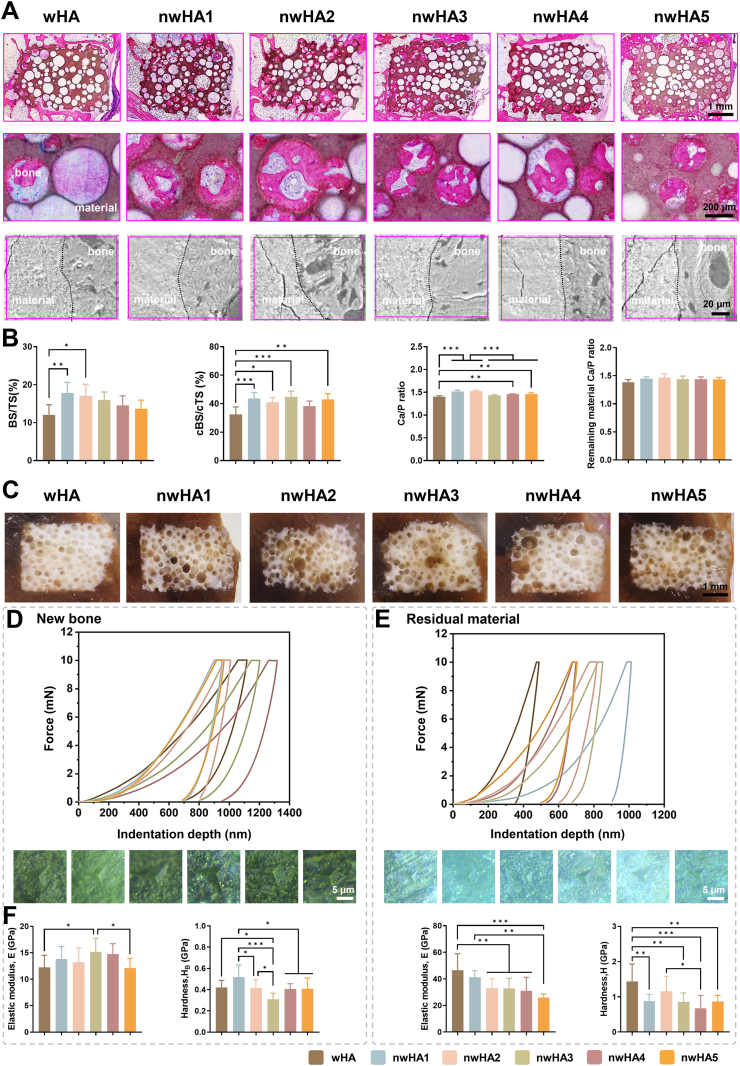
Histological and mechanical characterization of regenerated bone. (A) H&E-stained sections at 4 × and 20 × magnification, and SEM images showing bone–scaffold interfaces; scale bars: 1 mm (4 × ), 200 μm (20 × ), 20 μm (SEM). (B) Quantification of bone surface (BS/TS), contact surface (cBS/cTS) within 100 μm interface zone, and Ca/P ratios (EDS) in newly formed bone vs. residual scaffold; data as mean ± SD (n ≥ 8). (C) Optical imaging of PMMA-embedded undecalcified sections (B: bone, brown; M: material, white); scale bar: 1 mm. (D) Nanoindentation curves, microscopy images, and quantification of elastic modulus and hardness in newly formed bone; scale bar: 5 μm. (E) Corresponding mechanical analysis of undegraded material; all data as mean ± SD (n ≥ 8; ∗*p* < 0.05, ∗∗*p* < 0.01, ∗∗∗*p* < 0.001).

Undecalcified PMMA-embedded hard-tissue H&E sections demonstrated extensive bone ingrowth within the porous architecture of both uncoated wHA and nHA-coated scaffolds (Fig. 7A). nwHA1 and nwHA2 groups exhibited markedly deeper bone penetration from the defect margins into the central pores, forming continuous intraporal bridges and achieving tighter bone–scaffold contact compared with wHA controls. The mineralized tissue was closely interwoven with the ceramic framework, reflecting active remodeling and progressive osseointegration. In contrast, wHA sections frequently displayed cartilage-like lacunae at the interface, consistent with hybrid ossification involving chondrogenic intermediates. High-resolution SEM further confirmed seamless interfaces between regenerated bone and scaffold, with indistinct phase boundaries. Discrete nHA particulates were occasionally observed embedded within the neo-bone, suggesting incorporation of coating-derived particles into the forming mineral phase. Notably, in an exploratory pilot test, a sterile normal-saline suspension of detached nHA5 particles was injected into a 2-mm non-critical defect, and scattered mineralized bone islands were observed within the defect core at 8 weeks, suggesting that nHA5 particles may retain intrinsic osteoinductive activity even in the absence of a supporting scaffold (Supplementary Fig. S19). While this pilot experiment was not powered for quantitative comparison, it provides a plausible explanation for the more pronounced peri-implant remodeling in the nwHA5 group, consistent with a contribution from particle detachment/dissolution beyond interface-guided bridging.Energy-dispersive X-ray spectroscopy (EDS) mapping showed overlapping distributions of Ca, P, and O in mineralized regions, while carbon was enriched in adjacent soft tissue and osteocyte lacunae (Supplementary Fig. S20). Quantitative histomorphometry revealed that nwHA1 and nwHA2 supported significantly higher total new bone volume compared with wHA (Fig. 7B). Within the surgically defined 100 μm gap region—an established metric for early osseointegration—all nHA-coated groups except nwHA4 exhibited significantly greater bone filling than wHA, highlighting the enhanced integrative capacity of nHA coatings. Additionally, the Ca/P molar ratio of bone adjacent to nwHA1 and nwHA2 closely approximated the stoichiometric value of native hydroxyapatite (∼1.67), indicative of more advanced mineral maturation. Optical imaging of PMMA-embedded samples further showed darker staining in the nwHA1 group compared with wHA, consistent with higher mineral content (Fig. 7C). Tissue-level mechanical properties were evaluated by nanoindentation on the same PMMA-embedded specimens, with load–displacement curves analyzed using the Oliver–Pharr method (Fig. 7D and E). Newly formed bone in the nwHA groups displayed elastic modulus values closely matching those of host bone, indicating improved mechanical compatibility and load transfer. Among the groups, nwHA3-derived bone exhibited the highest modulus, while nwHA1-induced bone showed the highest hardness, in line with its greater mineral density. Notably, residual nwHA1 bioceramic displayed micromechanical properties well-matched to the adjacent neo-bone, demonstrating mechanical convergence at the interface. These histological, compositional, and micromechanical findings indicate that morphology-tailored nwHA scaffolds—particularly nwHA1 and nwHA2—promote superior bone regeneration, mineral maturation, and mechanical adaptation in osteoporotic defects, resulting in a robust interface capable of withstanding physiological loading.

### 2.9 Bone formation patterns and mineralization dynamics

**Fig. 8:**
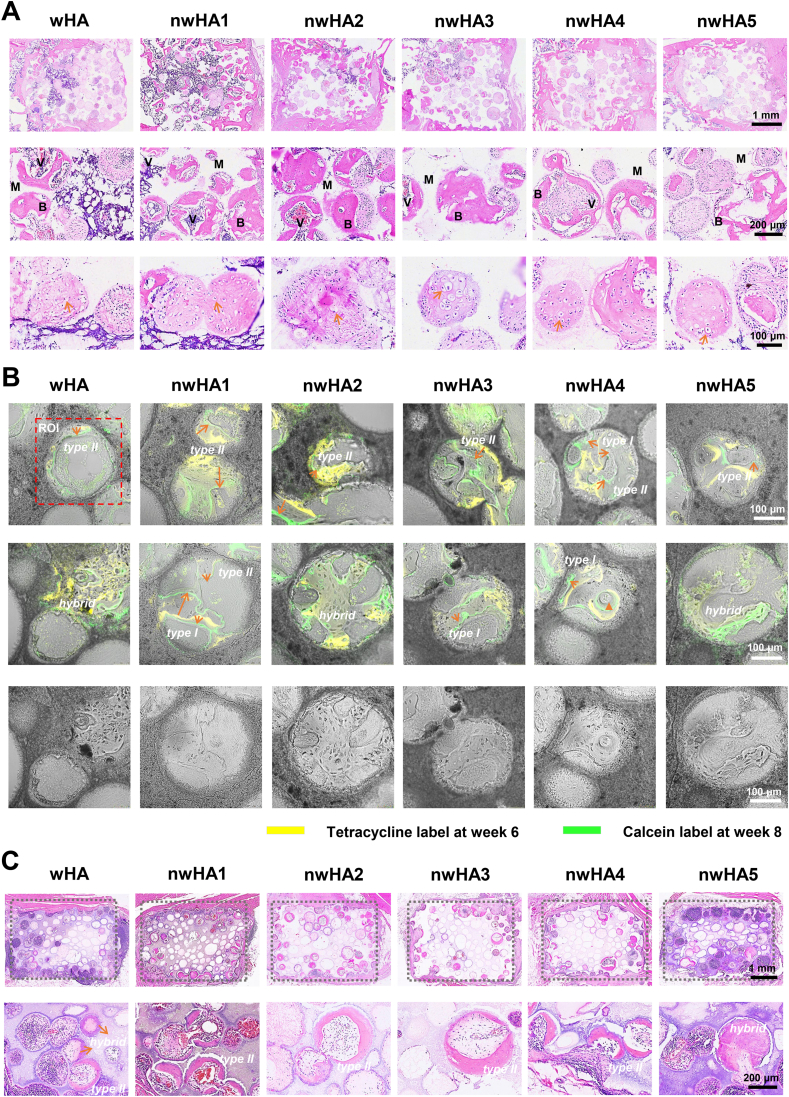
Ossification modes and mineralization dynamics in orthotopic and ectopic models. (A) Decalcified H&E-stained sections at 8 weeks, showing chondrocyte-like clusters at 4 × , 20 × , and 40 × magnification; scale bars: 1 mm (4 × ), 200 μm (20 × ), 100 μm (40 × ). (B) Sequential fluorochrome labeling (tetracycline: red, 6 weeks; calcein: green, 8 weeks) showing type I (wall-penetrating, bridging), type II (surface-appositional, concentric), and hybrid ossification with cartilage-like matrix features. The red dashed rectangle marks the regions of interest (ROI) used for mineral apposition rate (MAR) calculation, which evaluates the kinetics of mineralization by measuring the distance between the two fluorescent labels and dividing by the 14-day interval between the two injections. Scale bar: 100 μm. (C) H&E-stained sections of beagle dorsal muscle implants (12 weeks), demonstrating predominantly type II and hybrid ossification at 4 × and 20 × magnification; scale bars: 1 mm (4 × ), 200 μm (20 × ).

Decalcified soft-tissue H&E sections at 8 weeks post-implantation revealed distinct differences in intra-scaffold tissue organization across groups (Fig. 8A). nwHA1 and nwHA2 scaffolds displayed abundant osteoid- and bone-like tissue filling the pore interiors, accompanied by numerous embedded vascular lumen-like structures, indicative of active neovascularized bone formation. In contrast, wHA and nwHA5 defects showed sparse mineralized tissue, predominantly occupied by fibrous connective tissue. No persistent inflammatory infiltration was observed in any group, suggesting that the observed differences reflect regenerative capacity rather than chronic inflammation. To investigate ossification dynamics within scaffold pores, we first classified intra-scaffold bone formation into three recurrent patterns based on morphological and histological criteria: type I (wall-penetrating/bridging), type II (surface-appositional), and hybrid (multidirectional mineralization often accompanied by cartilage-like matrix) (Fig. 8B). Pattern assignment was independently performed by two blinded observers using a multimodal evaluation approach integrating undecalcified hard-tissue sections, decalcified histology, and fluorochrome-labeled CLSM imaging. The two observers showed substantial agreement (overall Cohen's kappa coefficient, κ = 0.674), with one-vs-rest Cohen's κ values of 0.752 (Type I), 0.611 (Type II), and 0.693 (hybrid). The distribution of ossification types across groups is summarized in Supplementary Fig. S21 nwHA1–nwHA4 scaffolds predominantly exhibited type I and type II patterns, while wHA and nwHA5 were more frequently associated with type II and hybrid patterns. These observations align with the more continuous mineral fronts and coherent bridging seen in fluorochrome-labeled sections from the former group. Mineral apposition rate (MAR), calculated from the inter-label distance between tetracycline (week 6) and calcein (week 8), served as a kinetic index of bone formation. Type I and hybrid regions exhibited significantly higher MAR than type II regions, with no significant difference between type I and hybrid. MAR data reinforce the functional heterogeneity among ossification modes and suggest that type II zones reflect slower or more surface-restricted mineralization. To further examine matrix composition, Safranin O/Fast Green and Toluidine Blue staining were applied to representative decalcified sections (Supplementary Fig. S22). Safranin O staining revealed aligned matrix strands extending into pores, with deep-green mineralized fronts suggestive of intramembranous ossification. In contrast, Toluidine Blue identified metachromatic zones and hypertrophic chondrocyte-like cells, indicating cartilage-involved remodeling, particularly in wHA and nwHA5 groups. In the ectopic muscle model (beagle dorsal muscle, 90 days), ossification was limited to type II and hybrid patterns (Fig. 8C). Although nwHA1–nwHA3 still supported some mineral deposition and vessel-like structures, type I bridging was virtually absent, consistent with the lack of mechanical loading and osteoprogenitor influx. Notably, nwHA5 showed disorganized mineral foci and mixed soft-tissue morphology, aligning with its hybrid-prone behavior under low-reactivity conditions. These results highlight that surface nanotopography shapes ossification geometry, but directional bridging (type I) additionally requires supportive host cues such as marrow-derived progenitors, vascular proximity, and interfacial mechanical stability.

## 3 Discussion

Osteoporotic bone defects, especially at load-bearing sites, remain difficult to heal. This challenge stems not only from low bone mass, but also from a hypo-responsive microenvironment characterized by impaired mechanosensitivity, dysregulated osteoimmune crosstalk, and delayed matrix remodeling [29,30]. Despite their compositional resemblance to bone mineral and inherent osteoconductivity, conventional HA bioceramics often underperform in osteoporotic defects due to poor early cellular engagement, leading to disorganized ingrowth and suboptimal long-term osseointegration [10,[31], [32], [33]]. Recent studies indicate that nanoscale topography can partially restore regenerative reactivity by reshaping adhesion-mediated cell behavior and downstream mechanotransductive programs [19,34,35]. Building on this, we developed a modular scaffold platform that decouples mechanical load-bearing from interfacial bioactivity. The wHA framework provides ∼4 MPa compressive strength, sufficient for non- or low-load-bearing osteoporotic defects (extension to segmental defects would require further strengthening). Morphology-specific nHA coatings deliver programmable nanoscale cues while preserving macroporous architecture. With macropore structure held constant, the divergent in vivo outcomes among nwHA variants highlight nanoscale geometry as a dominant regulator of regeneration quality. Aligned nanofiber (nwHA1) and nanorod (nwHA2) coatings promoted coherent intrapore tissue organization and superior osseointegration. By contrast, disordered nanoaggregates (nwHA5) yielded weaker intrapore osteogenesis but more pronounced peri-scaffold remodeling, suggesting stronger contributions from secondary particulate or ionic effects. Consistently, detached nHA5 particles induced scattered mineralized bone islands within the 2-mm defect core by 8 weeks, indicating a potential role for dispersed particle/ion-mediated signaling beyond interface-guided bridging. These findings suggest that effective osteoporotic bone repair relies more on early topography-mediated programming at the cell–material interface than on passive osteoconduction alone. Morphology-optimized nHA coatings thus offer a tunable strategy to overcome low regenerative reactivity in osteoporosis while preserving structural reliability.

Emerging evidence indicates that nanoscale topography can steer stem cell fate by reorganizing cell–matrix interactions and downstream mechanosensitive signaling [36,37]. Within this framework, the Hedgehog cascade has been increasingly recognized as a mechanotransductive node responsive to substrate geometry and stiffness, and disruption of this axis compromises Gli1^+^ osteoprogenitor expansion and mechanically driven bone formation [21,[38], [39], [40]]. Meanwhile, nHA morphology is a key determinant of cytocompatibility and osteoinductivity: rod- and sphere-like forms typically enhance osteogenic differentiation, whereas needle- or plate-like variants can increase cytotoxicity [[41], [42], [43], [44], [45]]. Our prior work identified rod-like nHA as a particularly effective morphology for improving osteoporotic osteoblast function and bone regeneration when applied as a surface coating on nwHA scaffolds [28,46]. Consistent with these insights, the present data support morphology-selective activation of a Hedgehog-centered program in MSCs. ECM-mimetic, anisotropic surfaces—particularly nwHA1 and nwHA2 coatings—exhibited strongest induction of canonical Hedgehog components (SHH/SMO/PTCH1/GLI1), with corresponding validation at the protein level for GLI1 and PTCH1 [[47], [48]]. This pattern argues for a pathway-specific response rather than a generic pro-osteogenic shift. Pharmacological inhibition with cyclopamine or siRNA-mediated GLI1 knockdown attenuated the scaffold-enhanced expression of osteogenic genes and dampened the accompanying pro-angiogenic signatures, suggesting that Hedgehog signaling functions upstream of an integrated osteoangiogenic program. Reciprocal knockdown of GLI1 and HIF-1α further revealed a reinforcing interaction between these factors, which aligns with the more coordinated bone–vascular integration observed for anisotropic morphologies, and is consistent with the early topography-dependent differences in MSC alignment quantified from F-actin images [33]. These results support a topography-responsive Hedgehog–HIF-1α module that couples osteogenesis with angiogenesis in osteoporosis.

Angiogenesis–osteogenesis coupling is essential for coordinated bone regeneration, particularly in metabolically compromised states such as osteoporosis, where reduced vascular perfusion and impaired paracrine signaling constrain repair [49,50]. In vitro, nwHA1 and nwHA2 scaffolds markedly enhanced angiogenic activity, as evidenced by accelerated HUVEC migration, robust branching tubulogenesis, and consistent upregulation of key angiogenic markers (CD31, VEGF, VWF, HIF-1α). HIF-1α, a mechanosensitive transcription factor that integrates hypoxia and matrix-derived biophysical cues, was consistently upregulated in both MSCs and HUVECs cultured on nwHA1 and nwHA2 surfaces [51,52]. Importantly, siRNA-mediated knockdown of HIF-1α in HUVECs selectively abrogated scaffold-induced expression of VEGF and other angiogenic genes while sparing basal levels, supporting HIF-1α as a central mediator of topography-driven endothelial activation. These results support a model in which nanofiber and nanorod topographies promote early cytoskeletal reorganization and focal adhesion signaling in both endothelial and osteogenic cells, thereby engaging HIF-1α-dependent transcriptional programs that coordinate vascularization with bone matrix formation [[53], [54]]. In contrast, the irregular aggregated morphology of nwHA5 elicited weaker angiogenic responses and failed to support coordinated intrapore osteogenesis, underscoring nanoscale structural order as a determinant of regenerative coupling. Consistent with these mechanistic readouts, in vivo implantation of nwHA1–nwHA3 elevated systemic PINP levels, a marker of active bone matrix synthesis [55]. Gait analysis further demonstrated functional recovery in the nwHA1 and nwHA2 groups, characterized by increased stride length and reduced base width, which closely paralleled micro-CT and histological regeneration outcomes [28]. These non-invasive functional metrics provide translationally relevant correlates of scaffold-mediated biomechanical improvement in osteoporotic bone repair.

Multi-scale analyses elucidated how morphology-specific nHA coatings orchestrate intra-scaffold bone regeneration, mineral maturation, and mechanical adaptation in osteoporotic defects. nwHA1 consistently delivered the most favorable regenerative outcome, characterized by accelerated intraporous bone ingrowth, enhanced trabecular organization, and near-stoichiometric Ca/P ratios (∼1.67) at the interface—hallmarks of advanced mineral maturation and seamless scaffold–matrix integration [15,56]. Nanoindentation further revealed that regenerated bone in nwHA1 achieved hardness values comparable to those of native cancellous bone, indicative of denser mineral packing and superior collagen–mineral organization that confer functional competence [57,58]. This mechanical convergence aligns closely with our prior observations in beagle segmental defects, where similar micro-/nanostructured interfaces enabled regenerated bone to progressively match host tissue properties despite differences in initial scaffold stiffness [22]. Comparable topography-driven remodeling has been reported in functionalized HA systems, underscoring the broader capacity of nanoscale cues to guide functional tissue restoration [33]. In contrast, nwHA5 restricted intrapore osteogenesis while promoting peri-scaffold trabecular remodeling—likely mediated by secondary ionic or particulate signaling—highlighting spatially divergent effects governed primarily by surface order rather than degradation rate alone. All coated groups exhibited controlled degradation without burst release, reinforcing that interfacial morphology is the dominant driver of regenerative performance in this system. All coated groups exhibited controlled degradation without burst release, reinforcing that interfacial morphology is the dominant driver of regenerative performance in this system. In this context, the sustained mineral maturation together with the moderate degradation profile of nwHA1 is well aligned with the intended non- or low-load-bearing osteoporotic defect setting without necessitating rapid scaffold resorption [57]. As a limitation, although the OVX rat is a widely used surrogate for estrogen-deficiency-driven bone loss, it only partially recapitulates the etiologic heterogeneity, species-specific remodeling differences, and scale-dependent biomechanics of human osteoporosis. Accordingly, our findings should be interpreted as proof-of-concept for topography-enabled rescue of a low-reactivity regenerative niche rather than as a direct predictor of clinical outcomes. The 8-week endpoint was deliberately chosen to capture early, topography-sensitive events while minimizing confounders from prolonged model duration and age-related changes. The reductionist monoculture assays were used to isolate cell-intrinsic responses to topographic cues.

The diversity of ossification patterns observed—along with enhanced vascularization in select groups—supports the view that surface nanotopography actively biases regeneration in osteoporotic bone, rather than acting as a passive osteoconductive scaffold. Integrating decalcified/undecalcified histology with sequential fluorochrome labeling, we identified three recurring intra-scaffold ossification modes: type I (wall-penetrating/bridging), type II (surface-appositional), and a hybrid mode marked by cartilage-like matrix alongside advancing mineral fronts. These modes showed marked spatial heterogeneity across pores and implantation sites, consistent with ossification in porous biomaterials occurring along a continuum rather than as strictly discrete pathways. In orthotopic defects, anisotropic topographies (nwHA1, nwHA2) favored type I and II ossification, enabling coherent pore bridging and mineral continuity—consistent with reports that hierarchical surfaces guide progenitor organization via integrin–cytoskeleton–FAK signaling [33,59]. By contrast, wHA and nwHA5 more frequently displayed type II and hybrid patterns, a shift plausibly reinforced by the ∼100 μm implantation-induced microgap, which can increase micromotion and weaken early tissue anchorage—effects exacerbated in osteoporotic bone with intrinsically reduced osteogenic responsiveness [28,46]. This context dependence was even more evident in ectopic implantation: despite their osteoinductive potential, nwHA1–nwHA3 scaffolds rarely induced type I bridging, suggesting that topographic guidance alone is insufficient without supportive mechanical and biological cues, consistent with our prior observations in bioactive biphasic calcium phosphate ceramics [23,24,60]. This orthotopic–ectopic divergence suggests that directional pore-spanning ossification depends on bone-site host inputs (marrow/periosteal progenitors, neovascularization, and local mechanical loading), which are limited in ectopic environments. Functionally, ossification mode was associated with tissue quality: hybrid (cartilage-involved) regions exhibited inferior micromechanical properties compared with type I– or type II–dominant regions, likely reflecting less mature mineral organization and residual unmineralized matrix, in line with established distinctions between endochondral and intramembranous programs [61,62]. Nonetheless, hybrid modes may confer an early advantage by enabling rapid matrix expansion prior to mineral maturation. Together, these findings indicate that nanotopography modulates the probability and geometry of de novo bone formation, whereas the dominant pathway is shaped by interfacial stability and site-specific microenvironmental permissiveness. Accordingly, further optimization may benefit from jointly tuning nanotopographical cues and the early mechanical milieu to preferentially support stable type I bridging and durable integration.

## 4 Conclusion

In summary, we engineered a mechanics–biology decoupled scaffold platform by selectively functionalizing wHA with morphology-specific nHA coatings. This design affords programmable nanotopographical control while fully preserving bulk porosity and mechanical competence. Among the five morphologies evaluated, the nanofiber-coated variant (nwHA1) consistently yielded the strongest performance in osteoporotic defects, manifesting superior bone ingrowth, augmented vascularization, and enhanced scaffold–host integration—outperforming both uncoated wHA and the other nHA-coated groups. Histological examination combined with sequential fluorochrome labeling revealed three principal intra-scaffold ossification modes—type I (wall-penetrating), type II (surface-appositional), and hybrid (cartilage-involved)—whose prevalence was governed by nHA morphology and the implantation milieu. These observations underscore the decisive influence of surface nanotopography in steering regenerative pathways. Mechanistically, nwHA1 selectively activated the canonical Hedgehog pathway (SHH–PTCH1–GLI1 axis) and upregulated HIF-1α expression in mesenchymal stem cells and endothelial cells alike, thereby orchestrating synergistic osteogenic and angiogenic responses. Crucially, pharmacological inhibition and siRNA-mediated knockdown confirmed that these responses were strictly topography-dependent and essential for the observed regenerative superiority. Collectively, our findings position nanoscale morphology as a tunable, bioinstructive parameter capable of overriding the subdued reactivity inherent to the osteoporotic microenvironment.

## 5 Experimental section

### 5.1 Synthesis of morphology-specific nHA

nHA with defined morphologies was synthesized via hydrothermal treatment using analytical-grade chemicals from Sigma-Aldrich (China), unless otherwise noted. The synthesis procedure for nHA is detailed below:

Fiber-like nHA (nHA1): A 1 L aqueous solution of 0.12 mol L^−1^ sodium phosphate (Na_3_PO_4_) was mixed with an equal volume of 0.20 mol L^−1^ calcium nitrate tetrahydrate (Ca(NO_3_)_2_·4H_2_O) under continuous stirring. The precursor solution was transferred to a Teflon-lined stainless-steel autoclave and hydrothermally treated at 120 °C for 4 h. The resulting precipitate was collected by centrifugation, washed thoroughly with deionized water, and dried at 60 °C for 24 h.

Short rod-shaped nHA (nHA2): A 1 L solution of 0.12 mol L^−1^ sodium dihydrogen phosphate (NaH_2_PO_4_) was mixed with an equal volume of 0.20 mol L^−1^ calcium chloride (CaCl_2_) under continuous stirring. The mixture was transferred to a Teflon-lined stainless-steel autoclave and hydrothermally treated at 120 °C for 2 h. The short rod-like nanoparticles were collected by centrifugation, washed thoroughly with deionized water, and dried at 60 °C for 24 h.

Primary nanoparticles-like nHA (nHA3): A 1 L solution of 0.12 mol L^−1^ sodium hexametaphosphate (Na_6_P_6_O_18_) was mixed with an equal volume of 0.20 mol L^−1^ calcium hydroxide (Ca(OH)_2_) under vigorous stirring. The mixture was transferred to a Teflon-lined stainless-steel autoclave and hydrothermally treated at 120 °C for 2 h. The precipitate was collected by centrifugation, washed thoroughly with deionized water, and dried at 60 °C for 24 h.

Raspberry-like spherical nHA (nHA4): A 1 L solution of 0.12 mol L^−1^ sodium hexametaphosphate (Na_6_P_6_O_18_) was mixed with an equal volume of 0.20 mol L^−1^ Ca(OH)_2_ under continuous stirring until homogeneous. The precursor solution was transferred to a Teflon-lined stainless-steel autoclave and hydrothermally treated at 120 °C for 2 h. The raspberry-like spherical nanoparticles were collected by centrifugation, washed thoroughly with deionized water, and dried at 60 °C for 24 h.

Corn-like aggregated nHA (nHA5): A 1 L solution of 0.12 mol L^−1^ Na_6_P_6_O_18_ was mixed with an equal volume of 0.20 mol L^−1^ Ca(OH)_2_ under vigorous stirring. The mixture was hydrothermally treated in a Teflon-lined stainless-steel autoclave at 150 °C for 4 h to form hierarchical corn-like aggregates. The product was collected by centrifugation, washed thoroughly with deionized water, and dried at 60 °C for 24 h.

### 5.2 Fabrication of nwHA bioceramic scaffolds

wHA bioceramics were prepared via a two-step process of precipitation and hydrothermal treatment. A 2 L solution of 0.85 mol L^−1^ Ca(NO_3_)_2_ (Sigma-Aldrich, China) was added dropwise to 2 L of 0.55 mol L^−1^ ammonium hydrogen phosphate ((NH_4_)_2_HPO_4_, Sigma-Aldrich, China) under stirring at 300 rpm, with the pH adjusted to 9.0 using 25% (w/v) aqueous ammonia and maintained throughout. After 10 h, the slurry was aged at room temperature for 48 h, washed three times with deionized water, and mixed with 30% hydrogen peroxide (H_2_O_2_) to induce foaming. The foamed slurry was molded, dried at 80 °C for 12 h, and sintered at 1100 °C for 2 h to form porous HA blocks. For surface activation, blocks were immersed in dilute nitric acid (pH 4.0) for 30 min, hydrothermally treated at 180 °C for 12 h in a 50 mL Teflon-lined stainless-steel autoclave, washed with deionized water, and dried at 60 °C for 24 h.

nwHA scaffolds were fabricated by coating wHA substrates with morphology-specific nHA (nHA1–nHA5) using vacuum infusion. Each nHA was dispersed in deionized water at 20 mg mL^−1^, sonicated at 100 W for 45 min (KQ-600KDE, Kunshan Ultrasonic Instruments, China) for uniform dispersion. The wHA scaffolds were placed in 250 mL Erlenmeyer flasks, evacuated at −0.08 MPa for 3 min to remove air from pores, and infiltrated with 50 mL nHA slurry under vacuum for 6 min to ensure conformal coating. The coated scaffolds were dried at 60 °C for 2 h, yielding composite scaffolds with hierarchical topography integrating microscale whiskers and nanoscale features specific to each nHA morphology.

### 5.3 Material characterization

The crystalline phase of nHA powders was identified by X-ray diffraction (XRD; Empyrean, PANalytical, Netherlands) using Cu Kα radiation (λ = 1.5406 Å). Relative crystalline ordering was semi-quantitatively assessed using the empirical crystallinity index (Xc) based on the V112/300 method:

$$\text{Xc}=\left(I_{300}\;–\;V_{112/300}\right)\;/\;I_{300}$$

where I_300_ is the peak height of the (300) reflection and V_112/300_ is the minimum intensity of the valley between the (112) and (300) peaks (31–33° 2θ), determined after background subtraction using a consistent procedure across all samples. Xc is reported as a dimensionless index and was used to compare relative crystalline ordering among phase-pure HA samples [63].

Surface chemical functionalities were analyzed by Fourier-transform infrared spectroscopy (FTIR; Nicolet 6700, Thermo Fisher Scientific, USA) in the range of 400–4000 cm^−1^ using KBr pellets. Transmission electron microscopy (TEM; Tecnai G2 Spirit, FEI, Netherlands) was used to evaluate particle morphology, size distribution, and crystallographic features. Samples were dispersed in anhydrous ethanol by ultrasonication and drop-cast onto carbon-coated copper grids. Dynamic light scattering (DLS; Zetasizer Nano ZS, Malvern Instruments, UK) was employed to determine hydrodynamic diameter and polydispersity index (PDI) of nHA1–nHA5 particles. Zeta potential was measured in aqueous dispersions to assess surface charge and colloidal stability. All measurements were performed at 25 °C in triplicate after 10 min of ultrasonication in deionized water. The Ca/P molar ratio of nHA powders was determined by ICP-OES (SPECTRO ARCOS, Germany) after complete dissolution in hydrochloric acid. Ca^2+^ and P^5+^ concentrations were quantified, and the Ca/P ratio was calculated accordingly.

Surface morphology and hierarchical micro-/nanostructure of bioceramic scaffolds were examined using scanning electron microscopy (SEM; S-4800, Hitachi, Japan). Due to the high-aspect-ratio whiskers and complex 3D porosity, direct roughness measurements by AFM or optical profilometry were not feasible on the scaffolds. Therefore, dense ceramic analog discs (Φ12 mm × 2 mm) were prepared using identical sintering, whiskerization, and coating protocols. Atomic force microscopy (AFM) was performed in tapping mode (scan area: 5 × 5 μm^2^), with 5–8 random regions analyzed per disc (n = 3 discs per group). Average roughness (Ra) and root mean square roughness (Rq) were calculated using Gwyddion software. SSA was measured by nitrogen adsorption using the Brunauer–Emmett–Teller (BET) method (ASAP 2020, Micromeritics, USA). Scaffold pore architecture, including total porosity and average pore diameter, was assessed by micro-computed tomography (micro-CT; VivaCT 80, Scanco Medical, Switzerland) at a voxel resolution of 10 μm. Compressive strength of bioceramic cylinders (Φ6 mm × 10 mm) was measured using a universal testing machine (UTM; AGS-X, Shimadzu, Japan) under uniaxial compression at a crosshead speed of 0.5 mm/min. Early-stage scaffold dissolution was evaluated in 1 M Tris–HCl buffer (pH 7.4) at 37 °C using a scaffold-to-buffer ratio of 1 g/100 mL. At days 1, 2, and 3, 500 μL of supernatant was collected and replaced with an equal volume of fresh buffer to maintain constant volume. Calcium and phosphate concentrations in the supernatant were quantified by ICP-OES.

### 5.4 Cell culture and analysis on modified bioceramic scaffolds

Mouse bone marrow-derived mesenchymal stem cells (MSCs; Cyagen Biosciences, Guangzhou, China) and human umbilical vein endothelial cells (HUVECs; Noblebio, China) were used to evaluate cytocompatibility and bioactivity of bioceramic discs (Φ14 mm × 2 mm). Discs were placed in 24-well ultra-low attachment plates (Corning Inc., NY, USA) to minimize non-specific background adhesion. MSCs were seeded at 2 × 10^4^ cells/well in α-MEM (HyClone, USA), and HUVECs were seeded at appropriate density in DMEM (HyClone, USA). Both media were supplemented with 10% fetal bovine serum (FBS; Gibco, USA) and 1% penicillin–streptomycin (BioWhittaker, Belgium). Cultures were maintained at 37 °C in a humidified 5% CO_2_ incubator, with medium refreshed every two days. Cell proliferation on bioceramic discs was assessed on days 1, 3, and 5 using the Cell Counting Kit-8 (CCK-8; Dojindo, Japan). CCK-8 reagent was added and incubated for 2 h at 37 °C, followed by absorbance measurement at 450 nm using a microplate reader (Infinite M200, Tecan, Switzerland). For HUVECs, viability on day 3 was evaluated by live/dead staining with fluorescein diacetate (FDA) and propidium iodide (PI; Topbio Science, China). Cells were stained for 5 min, and fluorescence images were acquired by confocal laser scanning microscopy (CLSM; TCS SP5, Leica, Germany) at excitation/emission wavelengths of 488/520 nm (FDA) and 535/617 nm (PI). After 3 days of direct culture, MSCs were fixed with 4% paraformaldehyde for 15 min and permeabilized with 0.1% Triton X-100 for 5 min. F-actin was stained with FITC–phalloidin (Sigma-Aldrich, USA) for 30 min, and nuclei were counterstained with DAPI (Sigma-Aldrich, USA) for 5 min. Imaging was performed by CLSM (excitation/emission: 495/518 nm for FITC, 358/461 nm for DAPI) under standardized settings. Cytoskeletal organization and cell polarity were quantified using ImageJ (NIH, USA). The Feret angle (major axis orientation) of individual MSCs (n ≥ 10/group) was measured to assess alignment, and cell shape anisotropy was evaluated using the MaxFeret/MinFeret ratio.

Scaffold extracts were prepared according to ISO 10993-12 guidelines. Sterilized discs were incubated in serum-free α-MEM at 0.2 g/mL for 72 h at 37 °C. Supernatants were collected, filtered through a 0.22 μm membrane, and used as culture medium for MSCs. Cell viability and proliferation in extract media were assessed using live/dead staining (Calcein-AM/PI) and CCK-8 assays after 48 h of culture (results shown for days 1, 3, and 7). To evaluate osteogenic potential, MSCs were cultured on bioceramic cylinders (Φ6 mm × 2 mm) in osteogenic induction medium for 7 days. Alkaline phosphatase (ALP) and Alizarin Red S (ARS) staining were performed using commercial kits (Alfa Aesar, USA) according to the manufacturer's instructions. Staining intensity was semi-quantitatively analyzed using ImageJ.

### 5.5 siRNA-mediated knockdown of GLI1 and HIF-1α

To investigate the functional roles of Hedgehog and hypoxia signaling pathways, MSCs and HUVECs were transfected with small interfering RNAs (siRNAs) targeting GLI1 or HIF-1α (GenePharma, China). A non-targeting siRNA (siNC) was used as a negative control. Cells were seeded in 6-well plates and transfected at ∼70 % confluence using Lipofectamine 2000 (Invitrogen, USA) according to the manufacturer's protocol. Briefly, siRNAs and Lipofectamine 2000 were separately diluted in serum-free Opti-MEM (Gibco, USA), mixed, and incubated for 20 min at room temperature to form transfection complexes. The complexes were added to the cells for 6 h, after which the medium was replaced with complete culture medium. Twenty-four hours post-transfection, cells were detached, counted, and re-seeded onto bioceramic discs (Φ14 mm × 2 mm) at the same density as in direct contact assays (Section 5.4) for an additional 48 h of incubation prior to downstream analyses (gene expression, protein levels, etc.). Knockdown efficiency was confirmed by qRT-PCR analysis. All experiments were performed in triplicate unless otherwise stated.

### 5.6 Quantitative real-time PCR analysis

Total RNA was extracted from MSCs and HUVECs cultured on bioceramic discs for 48 h using RNAiso Plus (Takara, Japan) according to the manufacturer's protocol. RNA concentration and purity were assessed using a NanoDrop 2000 spectrophotometer (Thermo Fisher Scientific, USA). For each sample, 500 ng of total RNA was reverse-transcribed into cDNA using the PrimeScript RT Reagent Kit (Takara, Japan). qRT-PCR was performed on a ProFlex PCR System (Applied Biosystems, USA) using SYBR Green Master Mix (Takara, Japan) in 20 μL reaction volumes. Thermal cycling conditions were as follows: initial denaturation at 95 °C for 30 s, followed by 40 cycles of 95 °C for 10 s and 60 °C for 60 s. A melt curve analysis (65–95 °C, 0.5 °C/s increments) was conducted to confirm amplification specificity. Relative gene expression was normalized to β-actin and calculated using the 2^−^ΔΔCt method. In MSCs, the following osteogenic and signaling-related genes were analyzed: BMP2, ALP, RUNX2, SHH, SMO, PTCH1, GLI1, GLI2, TGF-β, and HIF-1α. In HUVECs, expression of angiogenic and pathway-related genes was evaluated: CD31, VEGF, VWF, HIF-1α, SHH, PTCH1, and GLI1. Primer sequences were provided in Supplementary Tables S3 and S4.

To examine the functional roles of GLI1 and HIF-1α, MSCs and HUVECs were transfected with target-specific small interfering RNAs (siRNAs; GenePharma, China) using Lipofectamine RNAiMAX (Invitrogen, USA) according to the manufacturer's instructions. siNC served as a negative control. Cells were transfected at approximately 70 % confluence in 6-well plates. Transfection complexes were formed in serum-free Opti-MEM (Gibco, USA) and added to the cells for 6 h, after which the medium was replaced with complete culture medium. Cells were incubated for an additional 18 h post-transfection before being detached and re-seeded onto bioceramic discs for 48 h prior to RNA extraction. Knockdown efficiency and downstream gene expression were assessed by qRT-PCR. In MSCs, expression of GLI1, HIF-1α, RUNX2, ALP, and other osteogenic markers was quantified (Supplementary Table S5). In HUVECs, GLI1, HIF-1α, VEGF, CD31, and VWF levels were measured as indicators of angiogenic response (Supplementary Table S6).

### 5.7 Western blot analysis

MSCs were cultured on bioceramic discs for 48 h and lysed in ice-cold RIPA buffer (Beyotime Institute of Biotechnology, China) supplemented with protease and phosphatase inhibitor cocktails (Roche, Switzerland). Total protein concentration was determined using a BCA Protein Assay Kit (Bio-Rad Laboratories, USA). Equal amounts of protein (30 μg per lane) were separated by 12% SDS–PAGE and transferred onto PVDF membranes (Millipore, USA) using a wet transfer system (Bio-Rad, USA) at 350 mA for 2 h. Membranes were blocked with 5% (w/v) non-fat dry milk in TBST (TBS containing 0.1% Tween-20) for 2 h at room temperature, followed by overnight incubation at 4 °C with primary antibodies (1:1000 dilution) prepared in TBST containing 5% BSA. The primary antibodies used were: anti-BMP-2 (HUABIO, #ER80602), anti-ALP (HUABIO, #ET1601-21), anti-HIF-1α (HUABIO, #HA721997), anti-GLI1 (HUABIO, #ET1702-85), anti-PTCH1 (Absin, #abs115174), and anti-GAPDH (Bioworld, #AP0063). After washing with TBST (3 × 10 min), membranes were incubated for 1 h at room temperature with HRP-conjugated secondary antibodies (Cell Signaling Technology, USA; 1:3000 dilution in TBST), including goat anti-mouse IgG-HRP (#7076), goat anti-rabbit IgG-HRP (#7074), or rabbit anti-goat IgG-HRP (#7075), according to the host species of the primary antibody. Protein bands were visualized using an enhanced chemiluminescence (ECL) detection kit (Thermo Fisher Scientific, USA) and imaged with a ChemiDoc MP Imaging System (Bio-Rad, USA). Band intensities were quantified by densitometric analysis using ImageJ software and normalized to GAPDH. For pathway interference experiments, MSCs and HUVECs transfected with siRNAs targeting GLI1 or HIF-1α (as described in Section 5.5) were seeded onto nwHA1 scaffolds and cultured for 48 h prior to protein extraction. The expression levels of GLI1, HIF-1α, OCN, and ALP (in MSCs), as well as VEGF, HIF-1α and CD31 (in HUVECs), were analyzed by Western blot using the same protocol outlined above.

### 5.8 Tube formation assay

To evaluate the pro-angiogenic priming effect induced by direct contact with different bioceramic surfaces while minimizing nonspecific background adhesion, a Matrigel-based tube formation assay was performed following HUVEC pre-culture on bioceramic discs. Pre-sterilized discs (wHA, nwHA1–nwHA5; 10 mm diameter) were placed in 24-well ultra-low attachment plates (Corning, USA) to reduce unintended cell attachment to the well bottom/walls. HUVECs were seeded directly onto each disc at 2 × 10^4^ cells/well in complete DMEM (10% FBS, 1% penicillin–streptomycin) and cultured for 48 h (37 °C, 5% CO_2_). Cells were then detached from the discs using 0.25% trypsin–EDTA, neutralized, counted, and re-seeded at 3 × 10^4^ cells/well into 96-well plates pre-coated with 50 μL/well of growth factor–reduced Matrigel (Corning, Cat. No. 356231, Lot No. ABW82704), which had been polymerized at 37 °C for 30 min. Tube formation was allowed to proceed for 4 h under standard culture conditions, and capillary-like networks were imaged at 10 × magnification using an inverted phase-contrast microscope (Nikon Eclipse TS100, Japan). For each well, three random fields were captured using identical imaging settings. Quantification of tube-like structures (total tube length, number of nodes, and number of meshes) was conducted using ImageJ (NIH, USA) with the Angiogenesis Analyzer plugin. All tube formation assays were performed using the same Matrigel lot to ensure consistency.

### 5.9 Scratch wound healing assay

Endothelial cell migration was assessed using a scratch wound healing assay in a semi-contact configuration to evaluate the influence of nearby bioceramic surfaces without direct physical contact. Six-well plates were pre-marked on the underside with five parallel reference lines (∼0.5–1 cm spacing) to ensure consistent imaging positions across time points. HUVECs were harvested with 0.25% trypsin–EDTA (Gibco, Thermo Fisher Scientific), collected by centrifugation, and resuspended in DMEM supplemented with 10% FBS and 1% penicillin–streptomycin at 5 × 10^5^ cells/mL. Cells were seeded at 2 mL/well (1 × 10^6^ cells/well) and cultured to 90–100% confluence at 37 °C in a humidified 5% CO_2_ incubator. A straight scratch was created perpendicular to the reference lines using a sterile 200 μL pipette tip, followed by three gentle washes with PBS to remove detached cells and debris. Pre-sterilized bioceramic discs (wHA, nwHA1–nwHA5; 3 mm diameter) were affixed vertically to the inner wall of each well using medical-grade silicone adhesive (Sylgard 184, Dow Corning) and positioned adjacent to—but not in contact with—the scratched region. Serum-reduced DMEM (1% FBS, 2 mL/well) was then added, and plates were incubated under standard culture conditions. Images were acquired at 0, 6, 12, and 24 h using an inverted phase-contrast microscope (Nikon Eclipse TS100, Japan) at 4 × magnification. Three fields per well were captured at the intersections of the scratch and the reference lines. Scratch area was quantified using ImageJ software with the Wound Healing Tool plugin. Percent wound closure was calculated as ((*A*_*0*_*−A*_*t*_)/*A*_*0*_) × 100, where *A*_*0*_ is the wound area at 0 h and *A*_*t*_ is the wound area at each time point. Each group was tested in triplicate.

### 5.10 Animal surgery and in vivo evaluation

All animal procedures were approved by the Institutional Animal Care and Use Committee (IACUC) of Sichuan University, China (Approval No. 20221031004), and were conducted in accordance with the National Institutes of Health Guide for the Care and Use of Laboratory Animals. Thirty female Sprague–Dawley rats (12 weeks old, 200–230 g; Chengdu Dashuo Experimental Animal Co., Ltd., China) underwent bilateral ovariectomy (OVX) via a midline abdominal incision under intraperitoneal anesthesia with pentobarbital sodium (2 mg/100 g body weight). The incision was closed in layers using 4-0 Vicryl sutures (Jinhuan, China). After a 6-week recovery period to allow osteoporotic changes to develop, bilateral femoral defect surgery was performed under the same anesthetic regimen. Briefly, a 1.0 cm lateral incision was made on each hindlimb to expose the distal femoral shaft, and a 3.0 mm critical-sized defect was created in each femur using a trephine drill under continuous saline irrigation.

Rats were randomly assigned to six groups (n = 5 rats per group; 10 defects per group) and received press-fitted scaffolds (wHA, nwHA1, nwHA2, nwHA3, nwHA4, or nwHA5) in both defects. Locomotor function was assessed at 2, 4, 6, and 8 weeks post-implantation using ink-based footprint analysis: hind paws were coated with non-toxic ink and animals were allowed to traverse a 1 m × 10 cm runway lined with white paper. Stride length and base width were quantified from the footprints. At 8 weeks, rats were euthanized by pentobarbital sodium overdose; femora were harvested and fixed in 10% neutral buffered formalin for subsequent analyses.

The osteoinductive potential of the bioceramic scaffolds was further evaluated in a large-animal model via intramuscular implantation in four healthy adult male beagle dogs (8–10 kg, 1–2 years old). Animals were housed under controlled conditions (22 ± 2 °C, 55 ± 5% humidity, 12 h light/dark cycle) with ad libitum access to food and water. Under intravenous anesthesia with sodium pentobarbital (1 mg kg^−1^), six intramuscular pockets per dog (bilateral paraspinal musculature; three pockets per side) were created by blunt dissection through ∼2 cm longitudinal skin incisions. Cylindrical scaffolds (Φ3 mm × 4 mm; wHA and nwHA1–nwHA5) were implanted as one scaffold per pocket, such that each dog received one scaffold from each material group (within-animal comparison). Implant locations were randomly assigned using a pre-generated allocation list and counterbalanced across left/right and cranial/caudal positions to minimize potential site effects. Adjacent pockets were separated by ≥ 10 mm to reduce inter-site interaction. Muscle and skin were closed in layers with 2–0 Vicryl sutures (Jinhuan, China). Postoperative care included intramuscular gentamicin (20,000 U·kg^−1^·day^−1^) for three consecutive days, with daily monitoring of general health and wound healing. At 90 days post-implantation, animals were euthanized by intravenous overdose of sodium pentobarbital (150 mg kg^−1^). Implant sites with surrounding muscle tissues were excised and fixed in 10% neutral buffered formalin at 4 °C for 48 h prior to histological processing. Across the four dogs, each material group had four independent implants (n = 4 scaffolds per material in total).

### 5.11 Analysis of bone turnover markers

Systemic bone turnover was assessed by collecting whole blood from the heart of rats under terminal anesthesia at 8 weeks post-implantation. Blood was clotted at 4 °C for 3 h, then centrifuged at 1000×*g* for 15 min at 4 °C to isolate serum, which was stored at −80 °C. Serum levels of C-terminal cross-linked telopeptide of type I collagen (CTX-I, bone resorption marker) and procollagen type I N-terminal propeptide (PINP, bone formation marker) were quantified using ELISA kits (Uscn Life Science, Wuhan, China). Diluted serum (100 μL) was added to pre-coated 96-well plates, processed with detection reagents, and absorbance was measured at 450 nm using a microplate reader (BioTek Instruments, USA). CTX-I and PINP concentrations were determined from standard curves.

### 5.12 Micro-computed tomography analysis

Femoral specimens containing the implanted scaffolds were scanned using a high-resolution micro-CT system (VivaCT 80, Scanco Medical AG, Bassersdorf, Switzerland) at 70 kV and 114 μA, with an isotropic voxel size of 15 μm. Raw projection data were reconstructed into 2D cross-sectional images and rendered into 3D volumetric models using the Scanco evaluation software. Scaffold material and newly formed bone were segmented using a semi-automated, threshold-based method. A single global threshold value was determined by histogram-guided calibration and uniformly applied to all samples to ensure consistency across groups. Within the central defect region, intra-defect bone volume fraction (iBV/iTV, %) and bone mineral density of newly formed bone (iBMD, mg HA/cm^3^) were quantified. The residual material volume (RMV) was extracted from the segmented scaffold phase. The degraded scaffold volume (DV) was calculated as DV = MV – RMV, where MV represents the initial scaffold material volume prior to implantation. The bone substitution ratio (iBV/DV) was then computed to evaluate the coupling efficiency between scaffold degradation and new bone formation. To assess bone–implant integration, a 100 μm-wide concentric annular region of interest (ROI) was defined around the scaffold interface, within which the contact bone volume fraction (cBV/cTV, %) was measured. Additionally, a peri-defect trabecular ROI (1 mm × 1 mm × 2 mm; width × depth × height) adjacent to the scaffold–host bone interface was selected to evaluate local bone microarchitecture. The following trabecular parameters were quantified: bone volume fraction (BV/TV, %), bone mineral density (BMD, mg HA/cm^3^), trabecular number (Tb.N, mm^−1^), trabecular thickness (Tb.Th, μm), trabecular separation (Tb.Sp, μm), and bone surface–to–bone volume ratio (BS/BV, mm^−1^).

### 5.13 Histological and fluorescent labeling analysis

After micro-CT scanning, femoral specimens were processed for histological analysis. Each group consisted of 5 rats with bilateral femoral defects (left and right femora; n = 10 defects/group). To enable complementary analyses, the left femur from each rat was processed as undecalcified hard-tissue sections, while the right femur from the same rat was processed as decalcified soft-tissue sections. Left femora were dehydrated through a graded ethanol series (70–100%) and embedded in polymethyl methacrylate (PMMA). Sections were cut to 100–200 μm thickness using a precision microtome (SAT-001, AoLiJing, China), ground and polished to ∼50 μm, and stained with hematoxylin and eosin (H&E) to visualize newly formed bone and scaffold material. Sections were imaged at 4 × and 20 × magnification using a digital slide scanner (BA600, Motic, China). Adjacent unstained PMMA sections were sputter-coated with gold and examined by scanning electron microscopy coupled with energy-dispersive spectroscopy (SEM-EDS) to map elemental distributions (Ca, P, O, C) across the bone–implant interface. To assess mineralization kinetics, tetracycline (6 mg/kg) and calcein (30 mg/kg; Sigma-Aldrich, USA) were intramuscularly injected at 6 and 8 weeks post-implantation, respectively. Unstained PMMA sections (from left femora) were imaged by confocal laser scanning microscopy (CLSM; TCS SP5, Leica, Germany) at excitation/emission wavelengths of 405/580 nm (tetracycline, yellow) and 488/517 nm (calcein, green). The mineral apposition rate (MAR) was calculated as the average linear distance between the two labels divided by the 10-day labeling interval. After CLSM imaging, the same sections were gold-coated and re-examined by SEM-EDS.

Right femora (n = 5/group) and ectopic muscle–implant specimens (from beagle dogs and healthy rats) were decalcified in 10% EDTA (pH 7.2) for 4 weeks at room temperature, with solution changes every 3 days. After PBS rinsing, tissues were dehydrated, cleared in xylene, embedded in paraffin, and sectioned at 5–10 μm using a Leica rotary microtome (Germany). Sections were stained with H&E (all samples), Toluidine Blue, and Safranin O/Fast Green (for ectopic and selected femoral samples), and digitally scanned to evaluate host tissue response, ectopic bone formation, and ossification patterns. Ossification patterns were classified through a multi-modal, blinded evaluation. For each animal, five non-adjacent sections were selected, and three pores randomly chosen per section (n = 15 pores/animal; n = 5 animals/group). Two blinded observers independently scored each pore according to predefined criteria. Inter-rater agreement between the two blinded observers was quantified using Cohen's κ for the three-category classification, and disagreements were resolved by consensus for downstream analyses. Final classification integrated evidence from undecalcified PMMA sections (left femora) stained with H&E, decalcified paraffin sections (right femora and ectopic implants) stained with H&E, Safranin O/Fast Green, and Toluidine Blue and CLSM fluorochrome labeling with tetracycline and calcein (from left femora).

### 5.14 Immunohistochemical staining

Immunohistochemical staining was performed on paraffin-embedded sections (5 μm thickness) from 10-day ectopic muscle implantation samples to assess early expression of osteogenic and hypoxia-related markers (GLI1, BMP2, HIF-1α) and vascularization-associated proteins (CD31, endomucin). Sections were deparaffinized in xylene, rehydrated through a graded ethanol series, and subjected to heat-mediated antigen retrieval in 10 mM sodium citrate buffer (pH 6.0). Endogenous peroxidase activity was quenched with 3% hydrogen peroxide (H_2_O_2_) for 10 min, followed by blocking with 5% bovine serum albumin (BSA) for 30 min at room temperature. For osteogenic and hypoxia-related targets, sections were incubated overnight at 4 °C with primary antibodies against GLI1, BMP2, or HIF-1α (all at 1:300 dilution), followed by PBS washes and incubation with HRP-conjugated secondary antibody (1:500; Servicebio, China) for 50 min at room temperature. Immunoreactivity was visualized using a DAB substrate kit (BOSTER, China) and counterstained with hematoxylin. For angiogenesis-related markers, adjacent sections were incubated with primary antibodies against CD31 (OM287804, 1:100) and endomucin (OM251416, 1:100; Omnimabs, USA), followed by species-appropriate HRP-conjugated secondary antibodies. Signal development was performed using the same DAB kit, with light hematoxylin counterstaining for nuclear visualization. Positive staining appeared brown under light microscopy. Semi-quantitative analysis was conducted using ImageJ software. For each section, four peripheral regions (“four corners”) were imaged at 4 × magnification, and one central region of interest (ROI) was selected at 20 × magnification. Integrated optical density (IOD) of the positively stained areas was measured to evaluate relative protein expression levels across groups.

### 5.15 Nanoindentation analysis

To assess the mechanical properties of newly formed bone tissue adjacent to the implant interface, nanoindentation testing was performed using a nanoindenter (Nano Indenter G200, Agilent Technologies, USA) equipped with a Berkovich diamond tip. Femoral specimens embedded in PMMA were polished to a mirror finish using silicon carbide abrasive paper followed by a 0.05 μm alumina suspension to ensure a smooth and flat testing surface. Indentation was carried out under load control with a maximum load of 10 mN, a loading rate of 0.5 mN/s, and a 10 s hold period at peak load to minimize viscoelastic creep. The load and corresponding indentation depth were continuously recorded throughout the loading–unloading cycle to generate force–displacement curves. The elastic modulus (*E*) and hardness (*H*) were calculated using the Oliver–Pharr method based on the unloading segment of the curve. All materials were modeled as linear elastic, isotropic solids, assuming a Poisson's ratio of 0.3 for bone tissue. Six independent indentations were performed per sample within 100 μm of the implant interface in histologically defined regions, with a minimum spacing of 50 μm between adjacent indentations to avoid interaction effects.

### 5.16 Statistical analysis

Quantitative data are presented as mean ± standard deviation (SD). Statistical analyses were performed using GraphPad Prism (v9.3.0; GraphPad Software, San Diego, CA, USA). For comparisons among multiple groups, data were first evaluated for normality (Shapiro–Wilk test) and homogeneity of variance (Levene's test). If assumptions were satisfied, one-way analysis of variance (ANOVA) followed by Tukey's post hoc test was applied. When normality and/or homoscedasticity were violated, logarithmic transformation was performed; if assumptions remained unmet, the Kruskal–Wallis test with Dunn's multiple comparisons was used. All in vitro experiments included at least three independent biological replicates, and technical replicates were averaged within each biological replicate prior to statistical testing. For gait assessment in the bilateral femoral defect model, stride length and base width were quantified separately for each hindlimb using ink-based footprint tracking. Because both hindlimbs in each animal received the same scaffold type, each hindlimb was treated as an independent biological replicate (n = 10 per group). Longitudinal gait data were analyzed by two-way ANOVA with time and treatment as fixed factors, followed by Sidak's multiple comparisons test when appropriate. For siRNA knockdown validation, differences between two groups were analyzed using an unpaired two-tailed Student's t-test. Statistical significance was defined as p < 0.05 and denoted as ∗p < 0.05, ∗∗p < 0.01, and ∗∗∗p < 0.001.

## CRediT authorship contribution statement

**Rui Zhao:** Writing – original draft, Investigation, Formal analysis, Data curation. **Jiayi Chen:** Writing – original draft, Visualization, Investigation, Formal analysis. **Yongjia Li:** Writing – review & editing, Methodology, Investigation. **Hui Qian:** Validation, Software, Investigation, Data curation. **Xiangdong Zhu:** Visualization, Resources, Formal analysis. **Grazia Raucci Maria:** Resources, Methodology. **Luigi Ambrosio:** Visualization, Resources. **Xiao Yang:** Writing – review & editing, Supervision, Funding acquisition, Conceptualization. **Xingdong Zhang:** Supervision, Project administration, Conceptualization.

## Ethics approval and consent to participate

All experiments involving animals were conducted in accordance with the Guide for the Care and Use of Laboratory Animals from the National Institutes of Health. All procedures were approved by the Institutional Animal Care and Use Committee of Sichuan University (approval No. 20221031004).

## Declaration of competing interest

The authors declare that they have no known competing financial interests or personal relationships that could have appeared to influence the work reported in this paper.
