# Supplementary material for: Hierarchical micro-/nanostructured hydroxyapatite scaffolds promote osteoporotic bone regeneration via activation of hedgehog and HIF-1α signaling
Source: Bioact Mater. 2026 Feb 5;60:640–60. doi: 10.1016/j.bioactmat.2026.01.049 (PMC12919287; doi:10.1016/j.bioactmat.2026.01.049)
Supplement: Multimedia component 1 [file mmc1.docx]

Supporting Information

Hierarchical Micro-/Nanostructured Hydroxyapatite Scaffolds Drive Hedgehog- and HIF-1α-Mediated Osteoporotic Bone Regeneration

Figure S1. Hydrodynamic size distribution of morphology-specific nHA measured by dynamic light scattering (DLS). Representative DLS intensity curves of nHA1–nHA5 dispersed in deionized water, reflecting distinct size profiles corresponding to each nanostructure. Z-average diameter and polydispersity index (PDI) are summarized in Table S1 (mean ± SD, *n* = 3).

Figure S2. Quantitative assessment of scaffold macroporous architecture. Total porosity (%) and mean pore diameter (µm) of wHA and nwHA scaffolds measured by micro-computed tomography (micro-CT). Data are presented as mean ± SD (*n* = 5). Statistical analysis: one-way ANOVA with Tukey’s post hoc test or Kruskal–Wallis test with Dunn’s post hoc test, as appropriate; *p* < 0.05 indicates significance.

Figure S3. Specific surface area (SSA) of wHA and nwHA scaffolds. SSA was determined via nitrogen adsorption using the Brunauer–Emmett–Teller (BET) method. All nwHA groups showed significantly increased SSA compared to uncoated wHA. No significant differences were observed among the five nwHA subgroups. Data are mean ± SD (*n* = 4). Statistical analysis: Kruskal–Wallis test with Dunn’s post hoc test; *p* < 0.05 vs. wHA.

Figure S4. Early-stage ion release profiles of wHA and nwHA scaffolds. Cumulative calcium ion (Ca²⁺) release from cylindrical scaffolds after immersion in Tris–HCl buffer (pH 7.4, 37 °C) for 1 and 3 days. Data were quantified by inductively coupled plasma optical emission spectrometry (ICP-OES) and presented as mean ± SD (*n* = 3).

Figure S5. Cytoskeletal alignment and polarity of MSCs on bioceramic scaffolds. Feret angle distributions were quantified from phalloidin-stained MSCs cultured on wHA and nwHA scaffolds. nwHA1 and nwHA2 induced clustered orientations, indicating aligned cytoskeletal organization, whereas wHA and nwHA5 showed broad angular dispersion. Cell polarity was assessed using the MaxFeret/MinFeret ratio, with the highest anisotropy observed on nwHA1 and nwHA2, and the lowest on nwHA5. Analysis was performed using ImageJ (n ≥ 10 cells per group).

Figure S6. Extract-based cytocompatibility of MSCs. Live/dead fluorescence staining (Calcein-AM/PI) and CCK-8 assay of MSCs cultured in scaffold extracts for 48 h, prepared according to ISO 10993-12. All groups demonstrated high cell viability, with significantly enhanced proliferation in nwHA1 and nwHA2 extract groups compared to wHA. Scale bar = 200 µm. Data are shown as mean ± SD (*n* = 6). Statistical analysis: one-way ANOVA with Tukey’s post hoc test; *p* < 0.05 vs. wHA.

Figure S7. Osteogenic staining of MSCs cultured on wHA and nwHA scaffolds. Representative alkaline phosphatase (ALP) and Alizarin Red S (ARS) staining images of MSCs after 7 days of osteogenic induction. Semi-quantitative analysis revealed that nwHA2 induced the most prominent mineral deposition (ARS), while nwHA1 and nwHA5 showed relatively elevated early ALP activity. Quantification was performed using ImageJ. Data are shown as mean ± SD (n = 3); *p* < 0.05, Kruskal–Wallis test with Dunn’s post hoc.

Figure S8. qRT-PCR validation of GLI1 and HIF-1α knockdown in MSCs. MSCs were cultured on nwHA1 scaffolds and transfected with either siGLI1, siHIF-1α, or a non-targeting negative control siNC. qRT-PCR was performed to assess the knockdown efficiency of each siRNA relative to siNC. GLI1 expression was selectively suppressed in the siGLI1 group, while HIF-1α was reduced in the siHIF-1α group, confirming the specificity and effectiveness of each knockdown. Expression levels were normalized to β-actin using the 2⁻ΔΔCt method. Data are presented as mean ± SD (n = 6); p < 0.05 vs. siNC (unpaired two-tailed Student’s t-test).

Figure S9. qRT-PCR and Western blot validation of scaffold-induced osteogenic signaling and its inhibition by siGLI1/siHIF-1α. Representative immunoblots and densitometric analysis of ALP, RUNX2, OCN, GLI1, and HIF-1α in MSCs cultured on wHA, nwHA1, nwHA1 + siGLI1, and nwHA1 + siHIF-1α. Knockdown of either GLI1 or HIF-1α suppressed nwHA1-induced upregulation of osteogenic proteins.

Figure S10. HUVEC viability on wHA and nwHA scaffolds. HUVEC proliferation and metabolic activity were evaluated by CCK-8 assay after 1, 3, and 5 days of direct culture on wHA and nwHA bioceramic discs. nwHA1 and nwHA2 groups showed enhanced proliferation compared to wHA. Data are presented as mean ± SD (n ≥ 8). *p* < 0.05 by one-way ANOVA with Tukey’s post hoc test.

Figure S11. HIF-1α–dependent angiogenic activation in HUVECs stimulated by nwHA1 scaffolds. qRT-PCR and Western blot analyses of HIF-1α and downstream angiogenic markers (VEGF, CD31, VWF) in HUVECs transfected with siHIF-1α or siNC, and then directly co-cultured with nwHA1 bioceramic discs. HIF-1α knockdown significantly reduced angiogenic gene and protein expression under nwHA1 stimulation, but had minimal effect under basal conditions (no-disc control), suggesting scaffold-specific, HIF-1α–mediated activation.

Figure S12. Time-dependent HUVEC migration in semi-contact co-culture with bioceramic scaffolds. Scratch wound healing assay images captured at 6 h and 12 h following injury, showing HUVEC migration in response to scaffold proximity. In this semi-contact configuration, pre-sterilized bioceramic discs (wHA, nwHA1–nwHA5) were positioned near—but not in direct contact with—the scratch zone, allowing assessment of paracrine migration cues without physical interaction.

Figure S13. Hedgehog pathway gene expression in HUVECs following scaffold exposure. qRT-PCR analysis of GLI1, PTCH1, and SHH in HUVECs after 48 h of direct co-culture with wHA or nwHA bioceramic scaffolds. GLI1 was modestly upregulated in the nwHA3 and nwHA5 groups, PTCH1 was highest in the wHA group, and SHH showed no significant differences across groups. These findings suggest limited and heterogeneous Hedgehog pathway activation in endothelial cells. Data are mean ± SD (n = 6). *p* < 0.05 vs. wHA by one-way ANOVA with Tukey’s post hoc test.

Figure S14. Longitudinal gait analysis in osteoporotic rats after scaffold implantation. Stride length and base width were measured at 2, 4, and 6 weeks post-implantation to assess locomotor recovery. nwHA1 and nwHA2 groups demonstrated longer stride lengths and narrower base widths over time relative to wHA controls, indicating improved gait coordination. Data are shown as mean ± SD (n ≥ 8). Two-way ANOVA (time × treatment) with multiple comparisons; *p* < 0.05.

Figure S15. Early-stage micro-CT imaging of bone–scaffold interface in osteoporotic rat femoral defects. 3D and 2D reconstructed micro-CT images at 1 week post-implantation, confirming uniform ~100 μm surgical gap, defining region of interest for early osseointegration and periscaffold bone formation.

Figure S16. Quantitative analysis of scaffold degradation and bone substitution efficiency. Micro-CT–based quantification of degraded material volume (DV), calculated as the difference between the initial scaffold volume (MV) and residual material volume (RMV) at the study endpoint. The bone substitution ratio (iBV/DV) was derived to evaluate the coupling efficiency between scaffold degradation and new bone formation within the defect region. Data are presented as mean ± SD (n = 10). Statistical analysis was performed using one-way ANOVA followed by Tukey’s post hoc test (*p < 0.05).

Figure S17. Early host response to ectopic intramuscular implantation at 10 days.
Representative H&E-stained sections of decalcified paraffin-embedded implants showing peri-implant cellular infiltration and early neovascularization at 10 days. Immunohistochemical staining of GLI1, BMP2, and HIF-1α demonstrated elevated expression in nHA-coated groups, with morphology-dependent differences across scaffold types. Semi-quantitative analysis was conducted by integrated optical density (IOD) measurement. Data are mean ± SD (n = 5); *p* < 0.05 by one-way ANOVA with Tukey’s post hoc test.

Figure S18. Immunofluorescent detection of CD31 and endomucin (Emcn) in ectopic muscle implants. Fluorescence co-staining of endothelial markers CD31 and Emcn in peri-implant regions at day 10 post-implantation. Quantification of CD31⁺Emcn⁺ double-positive area fraction showed increased angiogenic activity in nwHA groups, particularly nwHA1 and nwHA2. Data are shown as mean ± SD (n = 5). *p* < 0.05 by one-way ANOVA with Tukey’s post hoc test.

Figure S19. Bone formation induced by injection of an nHA5 particle suspension into a 2-mm non-critical defect. Representative histological sections show scattered, isolated “bone-island”–like mineralized tissue within the defect core (blue arrows), spatially separated from the surrounding host bone margin. High-magnification views confirm mineralized matrix with osteocyte lacunae, consistent with de novo bone formation induced by detached nHA5 particles.

Figure S20. Elemental composition at the bone–scaffold interface via SEM-EDS. Energy-dispersive X-ray spectroscopy (EDS) mapping of undecalcified PMMA sections depicting elemental distribution across the scaffold–bone interface. Calcium (Ca) and phosphorus (P) signals were enriched in scaffold and mineralized bone regions, while carbon (C) accumulation marked soft tissue and osteocyte lacunae; oxygen (O) was distributed throughout mineralized and soft matrices.

Figure S21. Ossification mode classification and associated mineralization dynamics. Distribution of ossification patterns—Type I (intramembranous-like), Type II (endochondral-like), and Hybrid—across scaffold groups, determined by blinded scoring (n = 5 animals/group) based on a combined analysis of: (1) undecalcified PMMA histology (H&E), (2) decalcified paraffin sections (H&E), and (3) CLSM-based fluorochrome labeling (tetracycline/calcein). Mineral apposition rate (MAR) was calculated from the linear distance between fluorescent labels divided by the 14-day interval. Data are mean ± SD (n = 20 regions of interest); p < 0.01, *p* < 0.001 vs. Type II by one-way ANOVA with Tukey’s post hoc test.

Figure S22. Safranin O/Fast Green and Toluidine Blue staining of representative sections. Safranin O/Fast Green highlights oriented matrix strands and focal mineralized fronts within pores, while Toluidine Blue shows localized metachromatic signals and scattered chondrocyte-like lacunae in Hybrid regions; staining contrast was moderate due to prolonged decalcification and archival preservation.

Table S1. Physicochemical characteristics of morphology-defined nHA variants (nHA1–nHA5). This table summarizes key physicochemical parameters of five nHA morphologies synthesized via tailored hydrothermal methods. Hydrodynamic particle size and polydispersity index (PDI) were determined by dynamic light scattering (DLS) in deionized water at 25 °C. Zeta potential was measured in aqueous dispersion. Ca/P molar ratios were quantified by inductively coupled plasma optical emission spectrometry (ICP-OES) after dissolution in dilute HCl. Relative crystallinity was estimated from X-ray diffraction (XRD) patterns using the V₁₁₂/₃₀₀ method.

| Sample | Morphology Description | DLS Size (nm) | PDI | Zeta Potential (mV) | Ca/P Molar Ratio (ICP) | Crystallinity (XRD) |
| --- | --- | --- | --- | --- | --- | --- |
| nHA1 | Nanofibers | 173.9 | 0.096 | –17.0 ± 0.6 | 1.61 | 0.27 |
| nHA2 | Nanorods | 67.1 | 0.182 | –12.7 ± 0.7 | 1.58 | 0.32 |
| nHA3 | primary nanoparticles | 3.13 | 0.047 | –13.2 ± 0.5 | 1.31 | 0.20 |
| nHA4 | Raspberry-like clusters | 97.6 | 0.093 | –8.4 ± 0.3 | 1.32 | 0.20 |
| nHA5 | Corn-like aggregates | 149.8 | 0.140 | –9.4 ± 0.3 | 1.43 | 0.20 |

Table S2. Surface and mechanical characterization of morphology-specific nHA coatings on nwHA scaffolds. Coating morphology and nanoscale dimensions were characterized from representative SEM and TEM images. Compressive strength was measured using a universal testing machine. Due to the porous 3D architecture and vertical topography of the scaffolds, surface roughness was assessed on dense ceramic analog discs fabricated using identical sintering, whiskerization, and nHA-coating protocols. Roughness measurements were obtained using atomic force microscopy (AFM) from 5–8 randomly selected regions per disc (n = 3 discs/group). Surface roughness is reported as average roughness (Ra) and root-mean-square roughness (Rq), expressed as minimum–maximum ranges across all measurements.

| Group | Morphology | Dimensions (nm) | Compressive strength (MPa) | Ra (μm) | Rq (μm) |
| --- | --- | --- | --- | --- | --- |
| nHA1 / nwHA1 | Nanofiber | 237.2 ± 47.3 × 17.8 ± 6.0 | 3.9 ± 0.2 | 0.05–0.38 | 0.06–0.47 |
| nHA2 / nwHA2 | Nanorod | 67.5 ± 16.2 × 17.3 ± 4.4 | 4.3 ± 0.2 | 0.04–0.16 | 0.05–0.21 |
| nHA3 / nwHA3 | Primary nanoparticles | ~9.4 ± 1.9 (diameter) | 4.1 ± 0.3 | 0.37–0.62 | 0.46–0.76 |
| nHA4 / nwHA4 | Raspberry-like sphere | 87.6 ± 7.0 (diameter) | 3.9 ± 0.4 | 0.03–0.25 | 0.04–0.35 |
| nHA5 / nwHA5 | Corn-like aggregate | 134.5 ± 18.0 × 46.3 ± 3.5 | 3.7 ± 0.3 | 0.30–0.70 | 0.40–0.94 |

Table S3. Primer sequences for qRT-PCR analysis of osteogenic and Hedgehog signaling genes in mesenchymal stem cells (MSCs).

| Target | Forward primer | Reverse primer |
| --- | --- | --- |
| BMP2  ALP  RUNX2  SMO  PTCH1  SHH  GIL1  GIL2  TGF-β  HIF-1α  β-ACTIN | CATGGGTTTGTGGTGGAAGTGG  GTGGAATACGAACTGGATGAGAAGG GAACCAAGAAGGCACAGACAGAAG  GCCCGCCGAGAGATTGTTTG  TCTTGGTGTTGGTGTGGATGATG  AGGACGGCCATCATTCAGAGGAG  TGCCACCAAGCCAACTTTATGTC  ACTGCCTACATATCAAAGCCATCAAG  GATTTGACCTGTTGCCTGTGTGAC  ACTTCTGGATGCCGGTGGTCTAG  CACGAAACTACCTTCAACTCC | CGCTGTTTGTGTTTGGCTTGAC  CATAGTGGGAATGCTTGTGTCTGG GGCGGGACACCTACTCTCATAC  TGAGGACCACGAACCAGACTAC  GTGACATTGCTGATGGAGGTGAG  CCCAGTCGAAACCTGCTTCCAC  CCGAGGAGGAGGAGGAGGAG  ATCTCCATGCCACTGTCATTGTTG  TCTGCTTGAAGGACTCCATGTTCTC  CCATGTCGCCGTCATCTGTTAGC  CATACTCCTGCTTGCTGATC |

Table S4. Primer sequences for qRT-PCR analysis of angiogenesis-related genes in human umbilical vein endothelial cells (HUVECs).

| Target | Forward primer | Reverse primer |
| --- | --- | --- |
| CD31  VEGF  vWF  HIF-1α  GIL1  SHH  PTCH1  GAPDH | GCCGCATATCCAAGGTCAGCAG  GTTGGTGCCCGCTGCTGTC  GCCGACTTCAACAGGAGCAAGG  AGCGCAAGTCCTCAAAGCACAG  AGCCTGAATCTGTGTATGAAACTGA CTAGTCCGTGTCTCTCCGT  GGCTGGCTGGCGTCCTG  ACCCAGAAGACTGTGGATGG | GAGCAGGACAGGTTCAGTCTTTCA  CAAACAAATGCTTTCTCCGCTCTGA  GCAGCACCGTGACGTGGATG  CATCAGTGGTGGCAGTGGTAGTG  CGTGGATGTGCTCGCTGTTG  ACTTGTCCTTACTACCTCTGAGTC  CATCATCCACACCAACACCAAGAG  GGACCGTCCACTGTCACTTT |

Table S5. siRNA sequences used for GLI1 and HIF-1α knockdown in MSCs. siRNAs targeting human GLI1 and HIF-1α were synthesized by GenePharma.

| Target | Sense Strand | Antisense Strand |
| --- | --- | --- |
| GLI1  HIF-1α  Negative control | GGAUGAAGGACCUUGCGUUTT  CCAGUUGAAUCUUCAGAUATT  UUCUCCGAACGUGUCACGUTT | AACGCAAGGUCCUUCAUCCTT  UAUCUGAAGAUUCAACUGGTT  ACGUGACACGUUCGGAGAATT |

Table S6. siRNA sequences used for GLI1 and HIF-1α knockdown in HUVECs. siRNAs targeting human GLI1 and HIF-1α were synthesized by GenePharma.

| Target | Sense Strand | Antisense Strand |
| --- | --- | --- |
| GLI1  HIF-1α | GCAAAUAGGGCUUCACAUATT CUCCCUAUAUCCCAAUGGATT | UAUGUGAAGCCCUAUUUGCTT UCCAUUGGGAUAUAGGGAGTT |
